# UCHL1-dependent control of hypoxia-inducible factor transcriptional activity during liver fibrosis

**DOI:** 10.1042/BSR20232147

**Published:** 2024-06-14

**Authors:** Amy Collins, Rebecca Scott, Caroline L. Wilson, Giuseppe Abbate, Gabrielle B. Ecclestone, Adam G. Albanese, Demi Biddles, Steven White, Jeremy French, John Moir, Wasfi Alrawashdeh, Colin Wilson, Sanjay Pandanaboyana, John S. Hammond, Rohan Thakkar, Fiona Oakley, Jelena Mann, Derek A. Mann, Niall S. Kenneth

**Affiliations:** 1 Newcastle Fibrosis Research Group, Biosciences Institute, Faculty of Medical Sciences, Newcastle University, Newcastle upon Tyne, U.K.; 2 FibroFind Ltd, FibroFind Laboratories, Medical School, Newcastle University, U.K.; 3 Department of Biochemistry, Cell and Systems Biology, Institute of Systems, Molecular and Integrative Biology University of Liverpool, U.K.; 4 Biosciences Institute, Faculty of Medical Sciences, Newcastle University, Newcastle upon Tyne NE2 4HH, U.K.; 5 Department of HPB and Transplant Surgery, Freeman Hospital, Newcastle Upon Tyne, U.K.

**Keywords:** deubiquitinase, DUB, hypoxia inducible factors, liver fibrosis, UCHL1

## Abstract

Liver fibrosis is the excessive accumulation of extracellular matrix proteins that occurs in most types of chronic liver disease. At the cellular level, liver fibrosis is associated with the activation of hepatic stellate cells (HSCs) which transdifferentiate into a myofibroblast-like phenotype that is contractile, proliferative and profibrogenic. HSC transdifferentiation induces genome-wide changes in gene expression that enable the cell to adopt its profibrogenic functions. We have previously identified that the deubiquitinase ubiquitin C-terminal hydrolase 1 (UCHL1) is highly induced following HSC activation; however, the cellular targets of its deubiquitinating activity are poorly defined.

Here, we describe a role for UCHL1 in regulating the levels and activity of hypoxia-inducible factor 1 (HIF1), an oxygen-sensitive transcription factor, during HSC activation and liver fibrosis. HIF1 is elevated during HSC activation and promotes the expression of profibrotic mediator HIF target genes. Increased HIF1α expression correlated with induction of UCHL1 mRNA and protein with HSC activation. Genetic deletion or chemical inhibition of UCHL1 impaired HIF activity through reduction of HIF1α levels. Furthermore, our mechanistic studies have shown that UCHL1 elevates HIF activity through specific cleavage of degradative ubiquitin chains, elevates levels of pro-fibrotic gene expression and increases proliferation rates. As we also show that UCHL1 inhibition blunts fibrogenesis in a pre-clinical 3D human liver slice model of fibrosis, these results demonstrate how small molecule inhibitors of DUBs can exert therapeutic effects through modulation of HIF transcription factors in liver disease. Furthermore, inhibition of HIF activity using UCHL1 inhibitors may represent a therapeutic opportunity with other HIF-related pathologies.

## Introduction

Liver fibrosis is characterised by the accumulation of extracellular matrix proteins in response to hepatic wound repair and chronic liver disease [1–3]. Activation or transdifferentiation of quiescent hepatic stellate cells (qHSC) to an activated (aHSC) myofibroblast state is well-established as a central driver of fibrosis in experimental and human liver injury [1–3]. The aHSCs are the scar-forming cells responsible for the excessive synthesis, deposition and remodelling of extracellular matrix proteins that underpins the development and progression of liver fibrosis. HSC transdifferentiation is a highly regulated epigenetic process that results in genome-wide changes in gene expression and profound alterations to the cellular proteome [1,2]. Identifying and functionally characterising the key molecular regulators of this transformation is vital to understanding the mechanisms that promote progression of chronic liver disease to its end stages of cirrhosis and/or liver cancer.

We have previously identified Ubiquitin C-terminal Hydrolase-L1 (UCHL1) as being highly induced during HSC activation [4]. UCHL1, a member of the UCH family of deubiquitinases (DUBs), is virtually absent in qHSCs; however its expression increases dramatically to very high levels in aHSCs, with an estimated 300-fold induction at the mRNA level [4]. Genetic deletion/depletion of UCHL1 or chemical inhibition of UCHL1 DUB activity is sufficient to reduce levels of fibrotic markers in liver, cardiac and lung fibrosis, demonstrating a key role for UCHL1 in promoting fibrosis in multiple organs [4–6]. However, the cellular substrates that UCHL1 acts on to promote the fibrotic phenotype remain elusive.

Several UCHL1 substrates have been described in tumour cells including Epidermal Growth Factor Receptor (EGFR), Transforming Growth Factor β (TGF-β), SMAD2, and importantly for our current study, Hypoxia Inducible Factor 1 α (HIF1α) [7–9]. Hypoxia Inducible Factor 1 (HIF1) is a ubiquitously expressed heterodimeric transcription factor composed of an oxygen-sensitive α subunit and a constitutively expressed beta subunit, also called aryl hydrocarbon receptor nuclear translocator (HIF1β/ARNT) [10,11].

Activation of HIF and hypoxia-dependent signalling cascades is emerging as a regulator of liver fibrosis [12]. Hepatocyte-specific deletion of HIF1α results in significant decreases of pro-fibrotic mediator levels, such as platelet-derived growth factors (PDGFs), fibroblast growth factors (FGFs) and other growth factors leading to reduced ECM deposition and a reduction of the fibrotic phenotype [13]. Conversely, targeted genetic deletion of negative HIF regulators such as prolyl-4-hydroxylase domain (PHD) and von Hippel–Lindau (VHL) proteins results in high HIF levels and increase hepatic steatosis and fibrosis [14,15]. Regions of hypoxia can develop in the liver after acute liver injury; however, increased HIF1α levels and activity are observed in diseased liver before the development of hypoxia, suggesting an oxygen-independent mode of HIF activation in chronic liver disease [16,17]. These genetic studies implicate a role for HIF signalling in the biology of the HSC.

HIF activity can be elevated through the upregulation of factors that disrupt HIF1α degradation, even in the presence of sufficient oxygen [18,19]. Deubiquitinating enzymes such as UCHL1 promote HIF1 activity under normoxic and hypoxic conditions by disrupting ubiquitin-mediated proteasomal degradation of HIF1α [9]. In this present study, we investigated if UCHL1 plays a role in regulating HIF1 activity during HSC activation, and examined if UCHL1 regulates the expression of hypoxia- and pro-fibrotic gene expression in a tractable cellular model. HIF1α levels were elevated in aHSC. Furthermore, genetic deletion or chemical inhibition of UCHL1 markedly reduced HIF1α levels under both normoxic and hypoxic conditions. Expression of UCHL1 elevated levels of hypoxia-responsive and pro-proliferative/fibrogenic gene expression in a HIF-dependent manner by specifically removing degradative ubiquitination from the HIF1α subunit. As a small molecule inhibitor of UCHL1 was able to suppress fibrogenesis in a human precision cut liver slice (hPCLS) model of fibrosis, targeting UCHL1/HIF may be an interesting strategy to explore towards the prevention of fibrosis progression in chronic liver disease.

## Materials and methods

### Isolation of primary HSC

Primary human HSCs were isolated from normal margins of surgically resected liver. Mouse HSC were isolated from normal livers of *UCHL1*^–/–^ or WT littermate controls. Mice were euthanised by cervical dislocation and the liver was then excised. In both human and mouse tissues, liver tissue was cut into small pieces and then digested with pronase and collagenase B (Roche) at 37°C with agitation for ∼30 min, then passed through a nybolt filter to create a cell suspension. The cell suspension was subsequently separated by an 11.5% Optiprep gradient (Sigma). HSCs were seeded onto plastic (Corning), cultured in Dulbecco’s modified Eagle’s medium (Life Technologies) supplemented with 16% fetal bovine serum, pyruvate, glutamine, penicillin, and streptomycin (Life Technologies) and maintained in an incubator at 37°C with 5% CO_2_. For human cells, freshly isolated (day 0) cells were considered quiescent and (day 10) cultures regarded as activated. All murine experiments were performed in Newcastle University and authors hold appropriate licences for work relating to all experiments and animal procedures were approved by local ethical review committee and the UK Home Office (As described in [4])

HEK293 cells were maintained in DMEM, supplemented with 10% FBS, glutamine, penicillin, and streptomycin (Life Technologies) and maintained in an incubator at 37°C with 5% CO_2._ HEK293-HRE-luciferase cells were a kind gift from Professor Sonia Rocha, Liverpool, and maintained in DMEM, supplemented with 10% FBS, 0.5 µg/ml puromycin, glutamine, penicillin, and streptomycin (Life Technologies) and maintained in an incubator at 37°C with 5% CO_2._

UCHL1 was inhibited using LND-57444 (Sigma Aldrich) at a final concentration of 50 μM unless otherwise indicated.

### DNA constructs, siRNA and transfections

pcDNA3-HA-HIF1α (Addgene #18949), pcDNA3-HA-HIF1α P402A, and P564A (Addgene #18955) were gifts from William Kaelin supplied by Addgene. pEBB. pEBB Flag VHL, pEBB-N-biotin-HIF1α, and pEBB-His-ubiquitin have been previously described [20]. Flag-HA-UCHL1 (Addgene # 22563) was a gift from Wade Harper, supplied by Addgene. pEBB-HA-UCHL1 and pEBB-N-biotin-UCHL1 was subcloned from Flag-HA-UCHL1.

A standard calcium phosphate transfection method was used for the introduction of plasmid DNA and siRNA into HEK293 cells. Briefly, HEK293 cells were plated into 10 cm dishes at a confluency of 30% 2 h before transfection. For each 10 cm plate a total of 5 μg of plasmid DNA was diluted to 439 μl in sterile water. Subsequently, 61 μl of a 2 M CaCl_2_ solution was added to the DNA mixture, followed by a 5-min incubation at room temperature. The DNA-CaCl_2_ mixture was then combined with an equal volume of 2× HEPES-buffered saline (HBS) and incubated for an additional 20 min. The DNA-CaCl_2_-HEPES precipitate was added dropwise, with subsequent incubation at 37°C overnight. Following this incubation, the transfection medium was replaced with fresh growth medium. Cells were harvested 48 h post-transfection. For RNAi transfections 10 μl of a 20 μM siRNA stock replaced the plasmid DNA. Scrambled and HIF1α siRNA sequences have been previously described [20].

### Hypoxia inductions

Cells were incubated at 1% O_2_ in an *in vivo* 300 hypoxia work station (Ruskin, U.K.). For hypoxic inductions, cell culture media was pre-equilibrated at 1% O_2_ prior to being added to cells.

### Cell lysis and immunoblotting

Cells were lysed for protein extracts, and RNA extraction in the work station to avoid re-oxygenation. Cells were lysed in RIPA lysis buffer and immunoblotted as described [21]. Antibodies used were human HIF1α (Clone 241809, R&D systems), rodent HIF1α (#14179, Cell Signaling Technologies), hydroxy-HIF-1α (Pro564) (#3434, Cell Signaling Technologies), VHL (#68547, Cell Signaling Technologies), ubiquitin (sc-8017, Santa Cruz), β-actin (AC-74, Sigma), HA (Clone 16B12, Covance), Flag (M2, Sigma), Biotin (sc-53179, Santa Cruz), UCHL1 (#3524, Cell Signalling Technologies).

### Ubiquitination assays

For ubiquitination assays, cells were lysed at room temperature under denaturing conditions (8 M urea, 50 mM Tris [pH 8.0], 300 mM NaCl, 50 mM Na_2_HPO_4_, 0.5% NP-40, 1 mM PMSF), supplemented with protease inhibitors (Roche) and ubiquitinated material was recovered by rotation with NiNTA-agarose (Invitrogen) washed 3× with lysis buffer and analysed by western blotting [20].

### Co-precipitation assays

Forty-eight hours following transfection, cells were harvested in cell lysis buffer (20 mM Tris pH 8.0, 150 mM NaCl, 1 mM EDTA, 1% Triton X-100, supplemented with protease and phosphatase inhibitors). Biotinylated HIF1α was purified with streptavidin agarose beads (Sigma) and precipitates analysed by Western blotting [20].

### Quantitative reverse-transcription PCR

Total RNA was isolated using the Peqgold Total RNA Isolation Kit (Peqlab) according to the manufacturer’s instructions. Reverse transcription with random and oligo(dT) primers and MMLV reverse transcriptase (Quanta Biosciences) was performed on 500 ng of total RNA.

Quantitative PCR data were generated using the following experimental settings: hold 50°C for 3 min; hold 95°C 10 min; cycling (95°C for 30 s; 58°C for 30 s; 72°C for 30 s with fluorescence measurement for 45 cycles). All values were calculated relative to maximum hypoxic induction and normalised to RPL13A levels using the Pfaffl method [22]. Each cDNA sample was assayed in duplicate and the results shown are averages derived from three or four biological repeats with error bars indicating the standard deviation.

Primers for human samples were: RPL13A sense 5′-CCT GGA GGA GAA GAG GAA AGA GA-3′, antisense 5′-TTG AGG ACC TCT GTG TAT TTG TCA A-3′; ANKRD37 sense 5′-GTC GCC TGT CCA CTT AGC C-3′, antisense 5′-GCT GTT TGC CCG TTC TTA TTA CA-3′; CAIX sense 5′-CTT TGC CAG AGT TGA CGA GG-3′, antisense 5′-CAG CAA CTG CTC ATA GGC AC-3′; COLA1 sense 5′-ATG TGC CAC TCT GAC TGG AA – 3′, ANTISENSE 5′-CTT GTC CTT GGG GTT CTT GC-3′; GLUT1 sense 5′-CTG GCA TCA ACG CTG TCT TC-3′, antisense 5′-GCC TAT GAG GTG CAG GGT C-3′; HIF1α sense 5′-CAT AAA GTC TGC AAC ATG GAA GGT-3′, antisense 5′-ATT TGA TGG GTG AGG AAT GGG TT-3′; αSMA sense 5′- ACC CAG CAC CAT GAA GAT CA-3′, antisense 5′- TTT GCG GTG GAC AAT GGA AG -3′; TGFβ sense 5′- CGT GCT AAT GGT GGA AAC CC-3′, antisense 5′- TCG GAG CTC TGA TGT GTT GA-3′; TIMP1 sense 5′- GTT TTG TGG CTC CCT GGA AC-3′, antisense 5′- GTC CGT CCA CAA GCA ATG AG- 3′; VEGF sense 5′-CCT GGT GGA CAT CTT CCA GGA GTA CC-3′, antisense 5′-GAA GCT CAT CTC TCC TAT GTG CTG GC-3′.

Primers for murine samples were Hif1a sense 5′- CCT GCA CTG AAT CAA GAG GTT GC-3′, antisense 5′- CCA TCA GAA GGA CTT GCT GGC T- 3′.

All DNA primers were synthesised by Integrated DNA Technologies.

### Proliferation experiment

Clonal cell lines were seeded into 96 well plates at a density of 2500 cells/well. The plates were incubated at 37°C and measured using 5% PrestoBlue® after either 24, 48, 72 or 96 h incubation. The gain was determined after 24 h and was subsequently used to measure all plates to allow for comparison between time points. Raw values were plotted to indicate cell growth/proliferation over time. The data were plotted using GraphPad PRISM and the area underneath the curve was quantified.

### Luciferase assays

Lysates for luciferase assay were prepared in 1× passive lysis buffer (Promega), 100 μl per well of a 24-well plate. About 10 μl of lysate was incubated with 50 μl luciferase reagent (Promega) and measured for 10 s using Lumat LB9507 (EG&G Berthold). Graphs represent raw RLUs readings from four independent experiments. Raw RLU values are presented, and statistical analysis performed using a one-way ANOVA with Dunnett’s multiple comparisons test. A value of *P*<0.05 was considered statistically significant.

## Results

### UCHL1-dependent control of HIF activity during liver fibrosis

**Figure 1:**
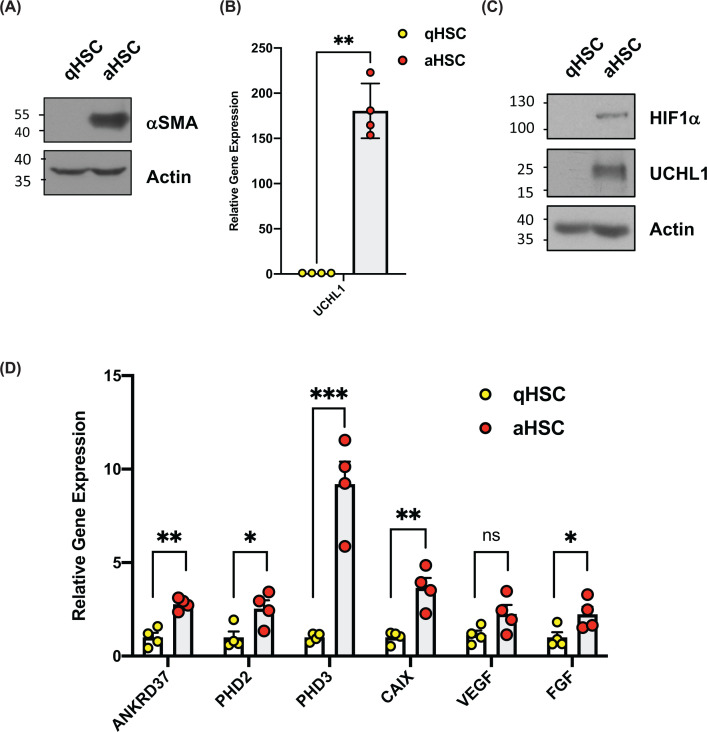
Expression of HIF1α and elevated HIF1 target gene expression in activated human hepatic stellate cells (HSCs) (**A**) Immunoblot analysis of lysates prepared from quiescent (Day 1) and activated (Day 10) HSCs. (**B**) Quantitative RT-PCR analysis of UCHL1 mRNA levels in from quiescent (Day 1) and activated (Day 10) HSCs All Relative expression compared with actin mRNA). (**C**) Immunoblot analysis of lysates prepared from quiescent (Day 1) and activated (Day 10) HSCs. (**D**) Quantitative RT-PCR of HIF1-target gene expression in quiescent (Day 1) an activated (Day 10) HSCs (All Relative expression compared to RPL13A mRNA). Quantitative RT-PCR was performed using four biological replicates. Statistical analysis was performed using GraphPad Prism by a unpaired Student’s *t*-test; *P≤0.05, **P≤0.01, ***P≤0.001.

The expression of UCHL1 is largely confined to brain and reproductive tissue in healthy individuals, however, aberrant expression of UCHL1 can be pathogenic in various disease states [8,24]. An example of this occurs during liver injury where UCHL1 transcript and protein expression is highly elevated during HSC transdifferentiation [4]. As UCHL1 can deubiquitinate and stabilise HIF1α in cancer cell lines, we sought to determine if there are changes in HIF1α levels during HSC transdifferentiation [9,25,26]. To investigate this, we analysed protein extracts and mRNA from undifferentiated, quiescent, primary human HSCs (qHSCs) and differentiated, culture-activated HSCs (aHSCs). As expected aHSCs expressed high levels of αSMA indicating they had transdifferentiated to the myofibroblast phenotype (Figure 1A). Consistent with our previous observations, UCHL1 was undetectable in qHSC and was induced at both the mRNA and protein level with HSC activation (Figure 1B,C). We next investigated if there were differences in HIF levels and activity between qHSCs and aHSCs. Immunoblot analysis revealed low or undetectable HIF1α protein expression in qHSCs which was elevated in aHSCs, despite these experiments being performed at 21% O_2_ (Figure 1C). To examine if the observed increase in normoxic HIF1α levels in activated HSCs correlates with increased HIF activity, we examined a panel of well-characterized HIF1α genes [20]. cDNAs prepared from RNA isolated from human qHSCs and aHSCs were used to quantitate the transcript levels of the hypoxia-responsive genes, ANKRD37, PHD2, PHD3, CAIX, VEGF and FGF [20]. Each of the HIF targets was elevated in the activated HSCs, consistent with elevated levels of HIF1α increasing HIF activity (Figure 1D).

### Genetic deletion or chemical inhibition of UCHL1 reduces HIF1α levels in hepatic stellate cells

**Figure 2:**
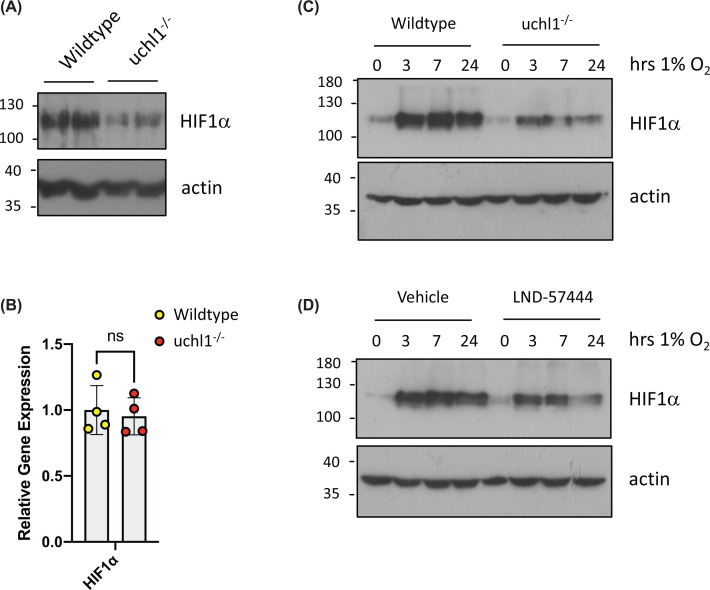
Reduction of HIF1α accumulation in UCHL1^−/−^ cells (**A**) Immunoblot analysis of lysates prepared from HSCs isolated from matched littermate wild-type and uchl1^−/−^ mice. (**B**) Quantitative RT-PCR analysis of Hif1a mRNA levels in from HSCs isolated from matched littermate wild-type and uchl1^−/−^ mice (all relative expression compared with RPL13A mRNA). Quantitative RT-PCR was performed using four biological replicates. Statistical analysis was performed using GraphPad Prism by a unpaired Student’s *t*-test; ns>0.05 . (**C**) HSCs isolated from matched littermate wild-type and uchl1^−/−^ mice exposed to 1% O_2_ for the indicated times. Whole-cell lysates (WCLs) prepared from these cells were subjected to immunoblot analysis to assess expression levels of the indicated proteins. (**D**) HSCs prepared from wild-type mice were pre-treated with LDN 57444 (uM) for 30 min before being exposed to 1% O_2_ for the indicated times. Whole-cell lysates (WCLs) prepared from these cells were subjected to immunoblot analysis to assess expression levels of the indicated proteins. Images presented are representative of three biological repeats.

To further investigate the functional relationship between UCHL1 levels and HIF activity in hepatic cells, protein extracts were isolated from HSCs derived from wildtype and UCHL1-deficient mice [27]. Consistent with UCHL1 being a positive regulator of HIF1α levels, UCHL1 deletion was associated with reduced levels of HIF1α protein expression under normoxic conditions (Figure 2A). In addition, UCHL1-dependent control of HIF1α expression appears to be post-transcriptional, as WT and *uchl1^−/−^* HSCs express similar levels of HIF1α mRNA (Figure 2B and Supplementary Figure S1). To determine if reduced levels of HIF1α in UCHL1-deficient HSCs persists under hypoxic conditions, cells were incubated at 1% O_2_ for various time points. In wild-type HSCs, there was an expected rapid and robust stabilization of HIF1α in response to hypoxic stress; however, hypoxia-dependent stabilization of HIF1α was reduced in later time points (7 and 24 h) in uchl1-deficient cells (Figure 2C).

As germline deletion of any protein has the potential to lead to unanticipated changes and adaptation, we studied the effects of small molecule UCHL1 DUB inhibitors on HIF1α levels. To this end we used LDN-57444 a reversible, competitive, active-site directed inhibitor of UCHL1 [28]. Murine aHSCs were pre-treated with LDN-57444 and exposed to hypoxia for the indicated times. Pre-treatment with the UCHL1 inhibitor attenuated HIF1α stabilization by low oxygen, consistent with UCHL1 preventing ubiquitin-mediated degradation of HIF1α (Figure 2D and Supplementary Figure S1).

### Elevated UCHL1 enhances HIF activity and increases expression of pro-fibrogenic genes

**Figure 3:**
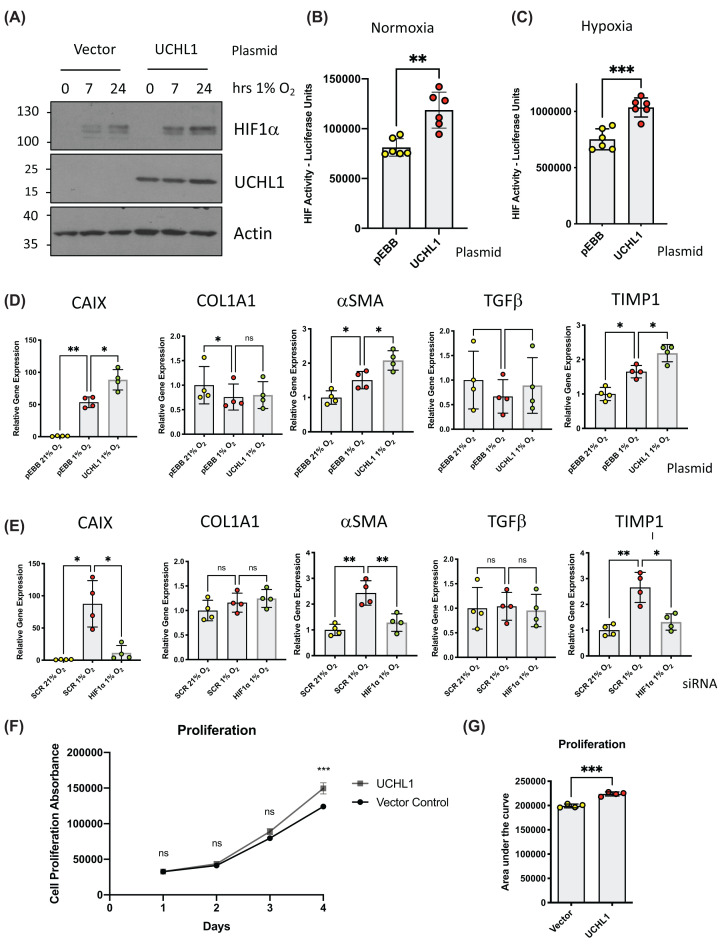
UCHL1 activates HIF-dependent gene expression (**A**) HEK293 cells transfected with the indicated plasmids before being exposed to 1% O_2_ for the indicated times. Whole-cell lysates (WCLs) prepared from these cells were subjected to immunoblot analysis to assess expression levels of the indicated proteins. (**B**) HEK293 cells stably expressing luciferase driven from the HRE promoter element (HRE-Luc) were transfected with UCHL1 and luciferase activity determined 48 h post-transfection. (**C**) as in (B) with cells incubated in 1% O_2_ 24 h prior to lysis. Luciferase assays was performed using six biological replicates. Statistical analysis was performed using GraphPad Prism by a unpaired Student’s *t*-test; *P≤0.05, **P≤0.01, ***P≤0.001. (**D**) Quantitative RT–PCR analysis of CAIX, COL1A1, aSMA, TGFb and TIMP1 mRNAs prepared from HEK293 cells transfected with the indicated plasmids before being exposed to 1% O_2_ for 24 h. (**E**) Quantitative RT–PCR analysis of CAIX, COL1A1, αSMA, TGFβ and TIMP1 mRNAs prepared from HEK293 cells transfected with the siRNAs before being exposed 1% O_2_ for 24 h Quantitative RT-PCR was performed using four biological replicates. Statistical analysis was performed using GraphPad Prism by a one-way ANOVA with Tukey’s multiple comparisons test; *P≤0.05, **P≤0.01, ***P≤0.001 (**F**) Proliferation of HEK293 cells transfected with empty vector or UCHL1 as measured by PrestoBlue. Graph displays Raw absorbance values. (**G**) The area under the curve Raw absorbance was calculated and analysed by unpaired students t-test. Proliferation assay was performed using 4 biological replicates. Statistical analysis was performed using GraphPad Prism by a unpaired Student’s *t*-test; *P≤0.05, **P≤0.01, ***P≤0.001.

To examine the role of UCHL1 in the expression of hypoxia-induced and pro-fibrogenic genes UCHL1 was expressed in HEK293 cells (a cell line that expresses no detectable levels of endogenous UCHL1) and exposed to hypoxia. Consistent with previous observations, expression of UCHL1 increased the levels of hypoxia-induced HIF1α compared with control cells as measured by immunoblot analysis (Figure 3A). HIF activity was assessed using HEK293 cells containing an integrated luciferase reporter construct possessing three copies of the hypoxia-responsive element (HRE) consensus-binding site. UCHL1 expression stimulated higher levels of luciferase activity under conditions of normoxia (Figure 3B) and hypoxia (Figure 3C) as compared to control cells, consistent with the UCHL1-dependent increase in HIF1α levels.

As expected, exposure to low oxygen resulted in an increase in expression of CAIX mRNA, a well-defined hypoxia-responsive target, which was further elevated with UCHL1 expression (Figure 3D). The hypoxia-dependent increase in CAIX expression was determined to be HIF1-dependent as hypoxia-induced CAIX expression was attenuated by siRNA-mediated depletion of HIF1α, consistent with CAIX being a HIF1-dependent target gene (Figure 3E).

To explore a role for UCHL1/HIF1α in the expression of pro-fibrogenic genes we then measured the transcript levels of COL1A1, αSMA TGFβ, and TIMP1, transcripts representing well-defined markers of fibrogenesis. Levels of αSMA and TIMP1 transcripts were also increased in hypoxic cells, however, no differences were seen for COL1A1 and TGFβ mRNAs (Figure 3D,E). Interestingly, depletion of HIF1α using specific RNAi prevented hypoxia-dependent increases in αSMA and TIMP1, suggesting that HIF1 activity is required for these hypoxia-associated changes in gene expression (Figure 3E). We examined the relationship between UCHL1 and hypoxia-induced expression of COL1A1, αSMA, TGFβ, and TIMP1 mRNAs. Of note, only the HIF/hypoxia-responsive pro-fibrotic genes, αSMA and TIMP1, were induced by UCHL1 overexpression, consistent with the model of UCHL1 increasing HIF1α levels and HIF activity (Figure 3D). To investigate the effect of UCHL1 on cellular growth, cell proliferation rates were compared between HEK293 cells expressing UCHL1 or transfected with vector alone (Figure 3F,G). HEK293 cells transfected with UCHL1 had a modest but significant increase in growth rates as measured by increased cell viability, consistent with a role for UCHL1 as a regulator of cell proliferation (Figure 3F,G).

### UCHL1 specifically deubiquitinates degradative ubiquitin chains from HIF1α

**Figure 4:**
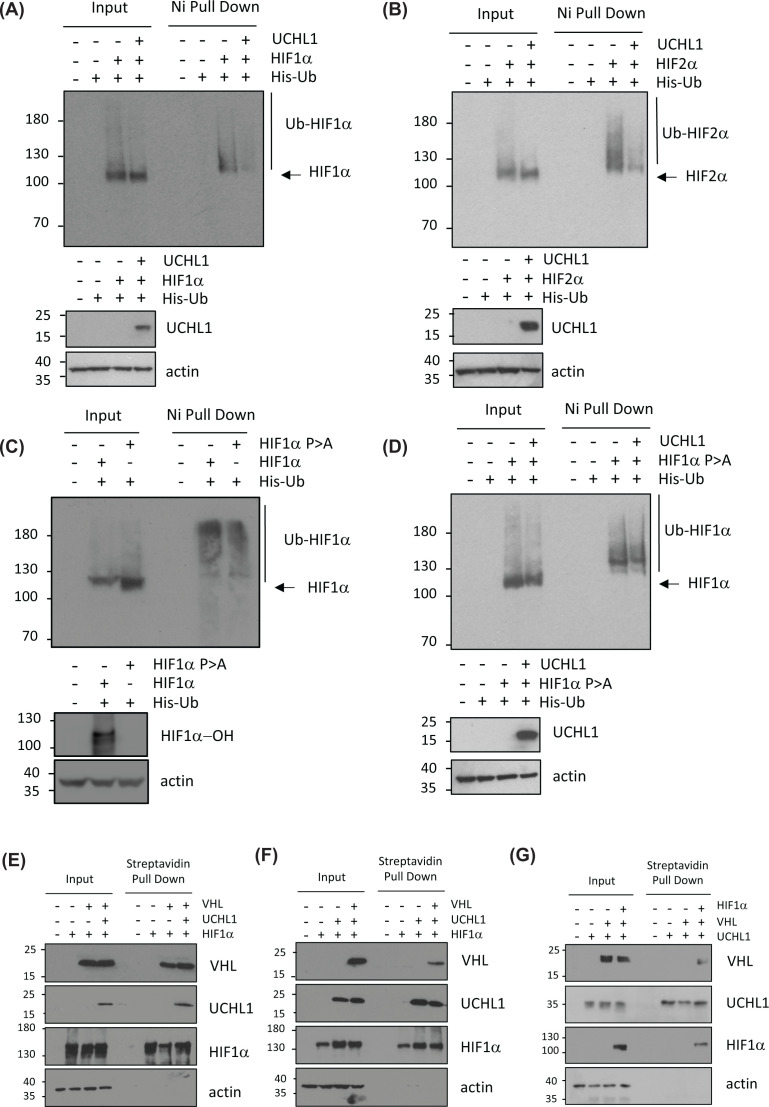
UCHL1 specifically degrades VHL-dependent Ubiquitin chains from HIF1α (**A**) HEK293 cells were transiently transfected with plasmids encoding HA- UCHL1, His tagged ubiquitin along with HA-HIF1α. Forty-eight hours post-transfection ubiquitinated complexes were stabilized by the addition of MG132 5 h prior to harvesting. Ubiquitinated material was recovered from lysates by incubation with nickel-coated beads and analysed by immunoblotting. UCHL1 expression was confirmed by immunoblotting input lysates with a anti UCHL1 antibody. (**B**) As in (A) using HA- HIF2α (**C**) HEK293 cells were transiently transfected with plasmids encoding HA- HIF1α, HA- HIF1α (P402A, P562A) with His-tagged ubiquitin. Ubiquitin pull downs performed as in (A). (**D**) As in (A) using HA-HIF1α (P402A, P562A) (**E–G**) HEK293 cells were transiently transfected with plasmids encoding (N-terminal Biotin Tagged) NBT- HIF1α, HA- HIF1α. NBT-UCHL1, HA-UCHL1 and Flag-VHL as indicated. Twenty-four hours prior to harvesting growth media was supplemented with biotin (4 μM). HIF1α and UCHL1 precipitates were recovered from lysates by incubation with streptavidin coated beads and analysed by anti HIF1α, anti-UCHL1, anti-Flag, anti-HA and anti-actin immunoblotting. Images presented are representative of three biological repeats.

When sufficient oxygen is available, HIF1α is destroyed by ubiquitin-mediated proteasomal degradation, mediated by the conjugation of lysine 48-linked ubiquitin chains to HIF1α by an E3 ubiquitin ligase complex containing VHL [29]. VHL-dependent regulation of HIF1α is well described; however, it is not the only E3 ubiquitin ligase that regulates HIF levels and activity. Indeed, an additional 17 E3 ubiquitin ligases have been reported to regulate the HIF pathway by various mechanisms, including regulation of subcellular location, macroautophagy and regulation of protein:protein interactions with co-activator/co-repressor proteins [30]. Indeed, unbiased mass spectrometry experiments reveal over 20 lysine residues on HIF1α are modified by ubiquitin in cells, but only three of these are required for VHL-dependent degradation (https://www.phosphosite.org/proteinAction.action?id=4987&showAllSites=true). To define the mechanistic relationships between UCHL1 and HIF, cells were co-transfected with tagged UCHL1, HIF1α and ubiquitin (Ub) expression vectors (Figure 4A). Despite the active site for UCHL1 appearing to limit access for proteins other than very short, ubiquitin-conjugated peptides, ubiquitination of HIF1α is markedly reduced when UCHL1 is co-expressed, consistent with results from previous studies [31] (Figure 4A). Interestingly, expression of UCHL1 also resulted in deubiquitination of HIF2α, indicating that UCHL1 can affect both HIFα isoforms targeted by VHL-dependent ubiquitination, thus exerting control of the two major HIFα subunits (Figure 4B). Further ubiquitination assays reveal that a HIF1α mutant that is resistant to VHL-dependent ubiquitination (HIF1α P402A, P564A) is still ubiquitinated in cells, albeit at lower levels (Figure 4C). However, overexpression of UCHL1 does not reduce levels of HIF1α P402/P564A ubiquitination, suggesting that UCHL1 only prevents the accumulation of degradation-inducing ubiquitin chains on to HIFα subunits (Figure 4D).

As analysis of UCHL1 structure suggests that it is unlikely to directly cleave ubiquitin chains from substrates; it has been proposed that UCHL1 decreases HIF1α ubiquitination by preventing the HIF1α/VHL interaction [9]. Co-precipitation experiments confirm that HIF1α precipitated efficiently with both VHL and UCHL1 (Figure 4E,F). However, we found that neither UCHL1 or VHL could displace the other from HIF1α, indicating that they can bind to HIF1α simultaneously through distinct interaction domains (Figure 4E,F). To confirm UCHL1 and VHL bind HIF1α simultaneously we investigated the UCHL1/VHL interaction in the presence or absence of HIF1α (Figure 4G). Only when HIF1α is present does VHL co-precipitate with UCHL1 indicating that HIF1α can simultaneously bind to UCHL1 and VHL (Figure 4G).

### UCHL1 inhibition reduces fibrosis in precision cut liver slices

**Figure 5:**
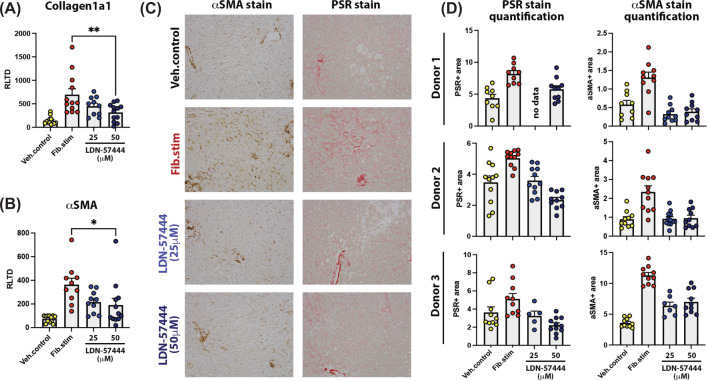
UCHL1 inhibitors have antifibrotic therapy in human liver slices (**A,B**) mRNA levels of Col1A1 and αSMA in human PCLSs at *t* = 72 h culture ± fib stim (TGFβ1/PDGFβ) ± LDN-57444 (96-h total culture). Data are mean ± SEM in *n* = 3 different donor livers. Statistical analysis was performed using GraphPad Prism by a one-way ANOVA with Tukey’s multiple comparisons test. *P≤0.05, **P≤0.01. (**C**) Images of αSMA and picrosirius red–stained human PCLSs from representative donor liver at *t* = 72 h culture ± fib stim (TGFβ1/PDGFβ) ± LDN-57444 (96-h total culture). (**D**) Graphs show percentage area of picrosirius red–stained or αSMA stained tissue in human PCLS of individual donors at *t* = 72-h culture ± fib stim (TGFβ1/PDGFββ) ± LDN-57444 (96-h total culture). Abbreviations: Cont, control; fib stim, fibrotic stimulation with TGFβ1/PDGFβ; Veh, vehicle.

We next asked if inhibition of UCHL1 effects fibrogenesis in a more physiological model of human liver fibrosis. To this end we selected human precision cut liver slices (hPCLS), which involves culturing intact slices of human liver tissue in a rocked bioreactor which maintains liver structure, cellular composition and metabolic activity for up to 6 days, offering a significant improvement on simple cell culture models [23]. Fibrosis was induced in hPCLSs by addition of transforming growth factor β1 (TGFβ1) and platelet-derived growth factor (PDGFβ) to hPCLS media. UCHL1 activity in hPCLS was blocked under these conditions using two different concentrations (25 and 50 μM) of LDN-57444. The higher concentration of LDN-57444 significantly inhibited TGFβ1+ PDGFβ stimulated increases in Collagen I and αSMA transcripts (Figure 5A,B respectively). Histological analysis of fibrogenesis in hPCLS was examined by αSMA and picrosirius red staining (PSR) which detects collagen. Exposure of hPCLS to TGFβ1+ PDGFβ increased hepatic myofibroblast activation (αSMA+ cells) and fibrogenesis denoted by increased PSR staining, with clear evidence of sinusoidal fibrosis in the latter stain, implicating αSMA+ aHSC as the major fibrogenic cells in this model. Studying effects across three independent donor human livers we observed that both concentrations of LDN-57444 suppressed the accumulation of αSMA+ fibrogenic cells while the higher concentration of the drug also attenuated deposition of fibrillar collagens (Figure 5C,D).

Our data collectively reveal that HIF1α expression and HIF1 activity are elevated in activated HSCs, even in conditions with adequate oxygen tensions. The high levels of UCHL1 expressed in activated HSCs specifically remove the degradative K48-linked ubiquitin chains from HIF1α, resulting in detectable HIF1α, even under normal oxygen tensions. The elevated HIF activity seen in HSCs results in elevated expression of pro-fibrogenic HIF- target gene expression in activated HSCs.

## Discussion

A role for HIF1α in the pathogenesis of fibrosis has been well documented, with scarred, hypoxic tissue evident in diseased liver [32]. However, increases in HIF levels and activity often precede acute hypoxic stress, therefore alternative modes of HIF activation may exist to activate HIF in fibrotic tissue [32]. HIF1α stability is primarily controlled through the addition of degradative ubiquitin chains by the PHD/VHL pathway to target HIF1α for proteasomal degradation However, HIF1α ubiquitination is reversible and ubiquitin chains can be removed through the action of deubiquitinating enzymes (DUBs) to exert fine control over HIF1α stability [9,25,26,33].

**Figure 6:**
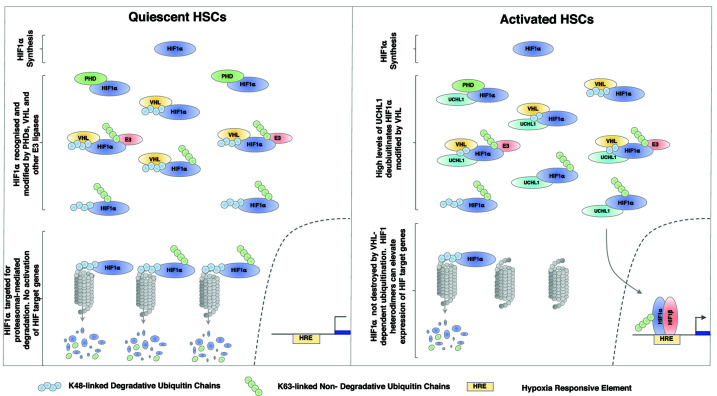
Model depicting the mechanism and physiological outcome of UCHL1-mediated deubiquitination of HIF1α in liver disease

In the present study, we describe a role for the UCHL1 DUB in controlling HIF activity in HSC under both normoxic and hypoxic conditions. Levels of UCHL1 were markedly elevated during HSC activation and this was associated with both increased stability of HIF1α and increased expression of HIF target genes (Figure 6). Genetic deletion or chemical inhibition of UCHL1 reduced HIF1α levels in activated HSCs under both normoxic and hypoxic conditions, thereby strongly implicating UCHL1 as an important regulator of HIF expression and activity (Figure 6).

HIF activity is mainly regulated by the sequential hydroxylation and ubiquitination of HIF1α and HIF2α by PHDs and VHL respectively; however, there are a number of E3 ubiquitin ligases and DUBs that can alter HIF stability, activity and sub-cellular localisation [33]. UCHL1 is one such DUB that has recently been shown to promote HIF1α deubiquitination in a variety of cancer cell types [9,25], although it is not clear if UCHL1 can deubiquitinate HIF1a directly due to restricted access of the active site resulting from the narrow channel formed by surrounding residues, this limiting substrate entry and influencing enzymatic activity [34].

UCHL1 is highly expressed in the brain, where it is involved in cell death, learning, and memory [34]. Increased expression of UCHL1 has been linked to neurodegeneration [24], cancer [8,35], and fibrosis-associated diseases [4,36,37]. Using small molecule inhibitors of UCHL1, we reveal that HIF signalling in myofibroblasts can be suppressed, potentially offering a therapeutic strategy to target HIF signalling in fibrotic tissue. In support of this idea, active fibrogenesis in a human pre-clinical model of human liver fibrosis was suppressed by UCHL1 inhibition (Figure 5). In this model, exposure of liver slices to the well-characterised autocrine stimulators of fibrosis, TGFβ1+ PDGFβ, robustly induces fibrogenesis, thus, pharmacological inhibition of UCHL1/HIF signalling in this model is highly relevant for translation to human liver disease. Interestingly our data indicate that only a subset of fibrotic genes are induced by overexpression of UCHL1 in cells, indicating that UCHL1 is necessary for fibrotic gene expression, but not sufficient (Figure 3D). This may be reflective of the different cell types or other factors in stellate cell activation. In addition, fibroblasts are now recognised as a highly heterogenous, dynamic plastic population of mesenchymal cells not only in the context of wound healing and fibrosis [38] but also in solid tumours, where distinct phenotypes of cancer-associated fibroblasts influence the biology of the evolving tumour microenvironment [39]. Hypoxic microenviroments in tumours and scar tissue along with high level expression of UCHL1 may therefore contribute to fibroblast diversity and functionality through targeted modulation of gene expression.

Recent work using an alternative UCHL1 inhibitor, IMP-1710, revealed a reduction of expression of profibrogenic markers in fibroblasts isolated from patients with idiopathic pulmonary fibrosis, demonstrating the importance of UCHL1 signalling in fibrosis [6]. This supports the concept of a broader role for UCHL1 in the control of fibroblast gene expression and phenotype. TIMP-1 is a major determinant of the stability of fibrotic matrix through its function as a broad specificity inhibitor of matrix metalloproteinases including MMPs, ADAMs and ADAMTs [40]. Hence, as we observed TIMP-1 to be under the positive regulation by UCHL1 and HIF1α this highlights potential for inhibition of UCHL1 to not only suppress fibrosis progression but to also reverse fibrosis, although the latter remains speculative.

In addition to the link between UCHL1 and HIF in promoting the expression of a subset of pro-fibrogenic genes, there is a clear link between UCHL1 and HIF activity in a variety of tumour types [9,25,26]. UCHL1 activity correlates with the poor prognosis of patients with breast and lung cancers, and a strong correlation between UCHL1 levels and HIF activity was observed in patient samples [9]. It would therefore be of great value to investigate the efficacy of UCHL1 inhibitors in treating cancer cell types with elevated UCHL1 and HIF activity.

## Supplementary Material

Supplementary Figure S1

## Data Availability

All data are available in the manuscript. Raw files for immunoblot analysis are provided in the supplementary information.

## Open Access

Open access for this article was enabled by the participation of University of Liverpool in an all-inclusive *Read & Publish* agreement with Portland Press and the Biochemical Society under a transformative agreement with JISC.

## Abbreviations

DUB: deubiquitinase
HIF: hypoxia-inducible factor
HSC: hepatic stellate cell
UCHL1: ubiquitin C-terminal hydrolase-L1
