# Supplementary material for: UCHL1-dependent control of hypoxia-inducible factor transcriptional activity during liver fibrosis
Source: Biosci Rep. 2024 Jun 14;44(6):BSR20232147. doi: 10.1042/BSR20232147 (PMC11182734; doi:10.1042/BSR20232147)
Supplement: Supplementary Figure S1 [file BSR-2023-2147_supp.pdf]

Full Scans
Figure 1A

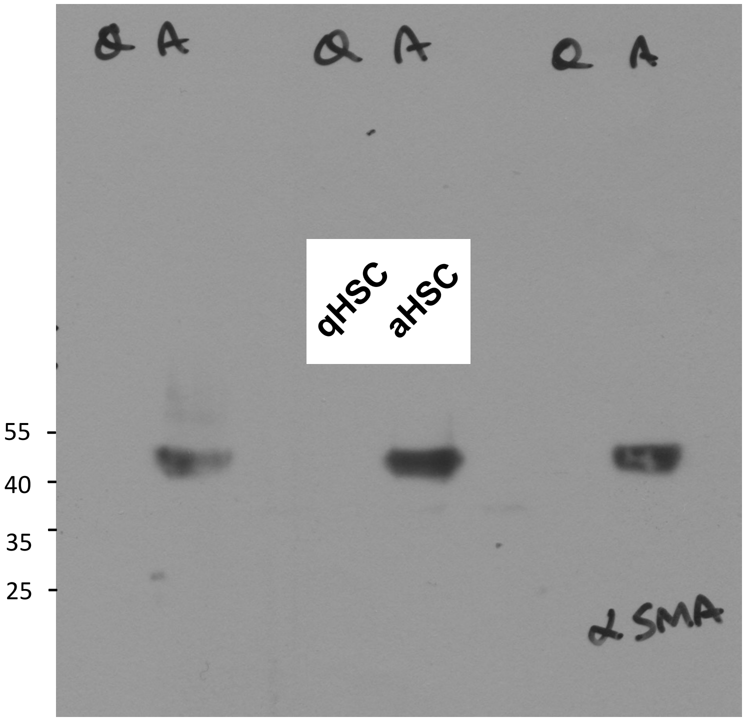

αSMA

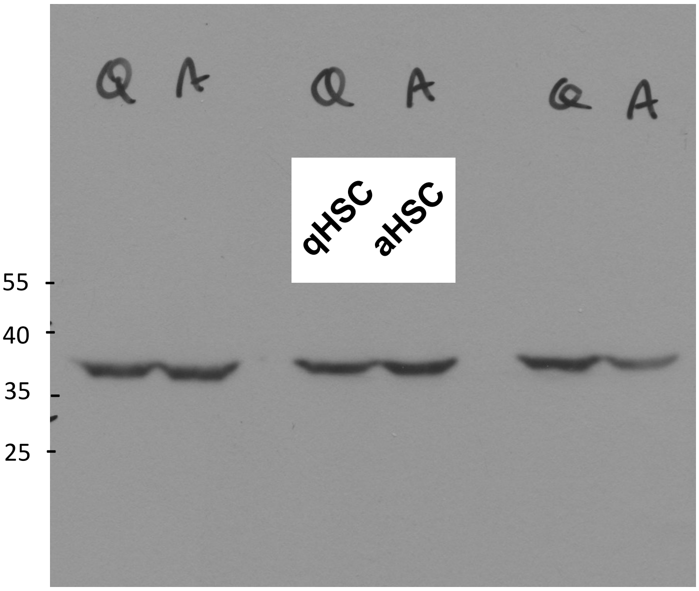

Actin

Full Scans
Figure 1C

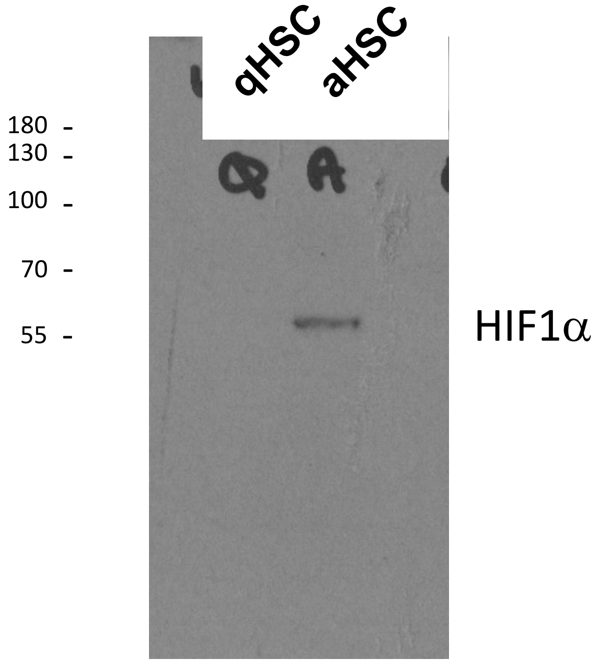

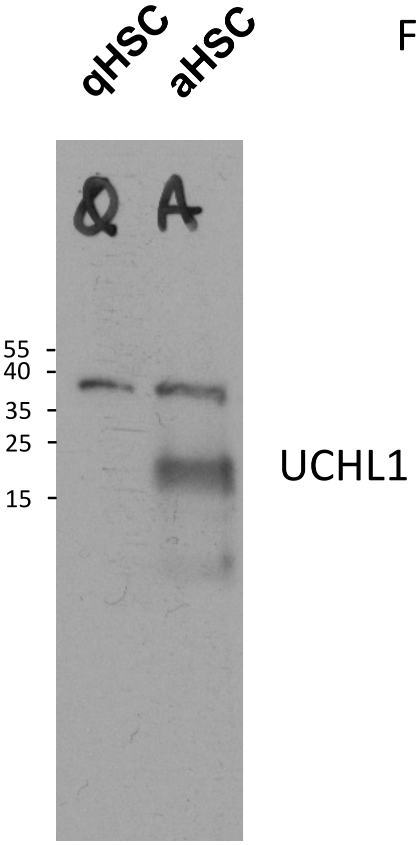

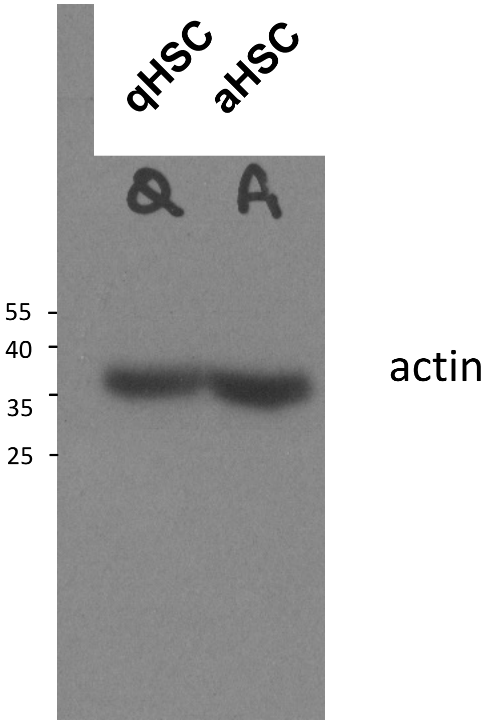

Full Scans
Figure 2A

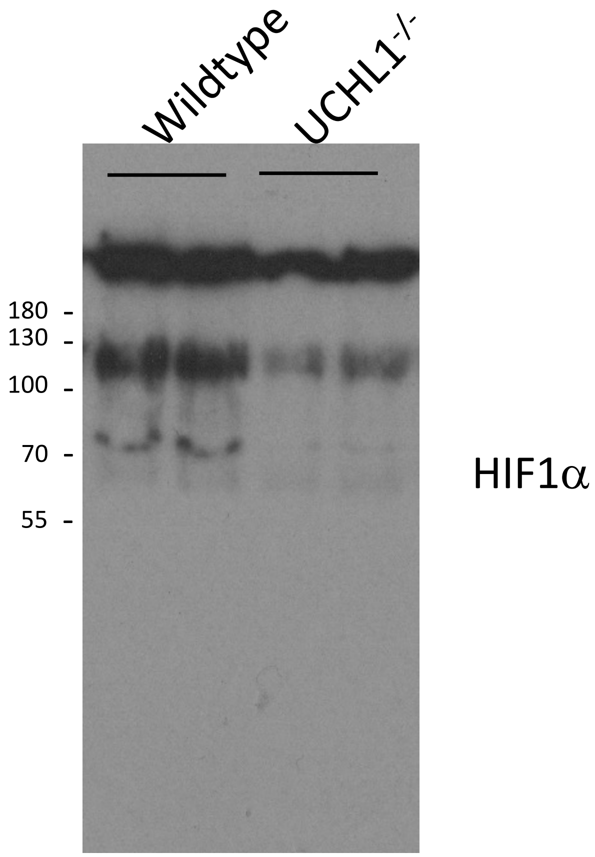

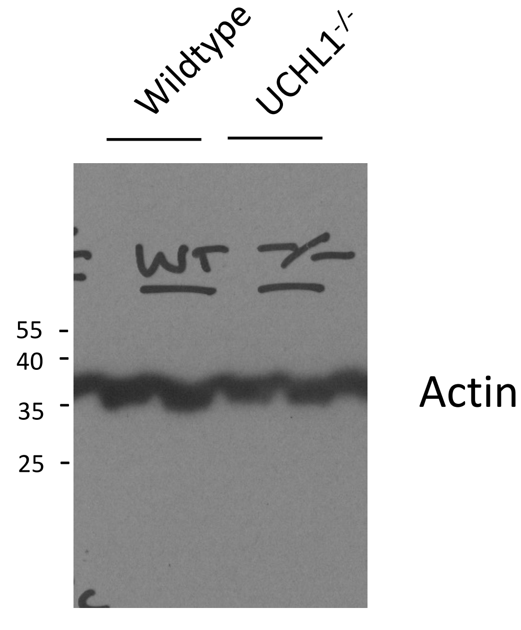

Full Scans
Figure 2C

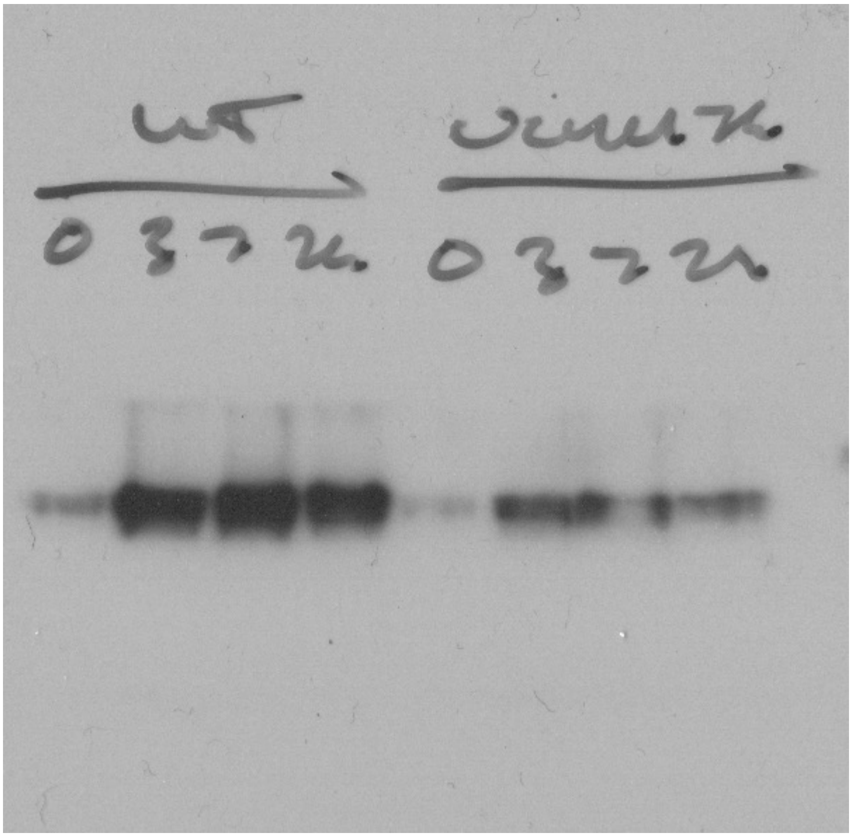

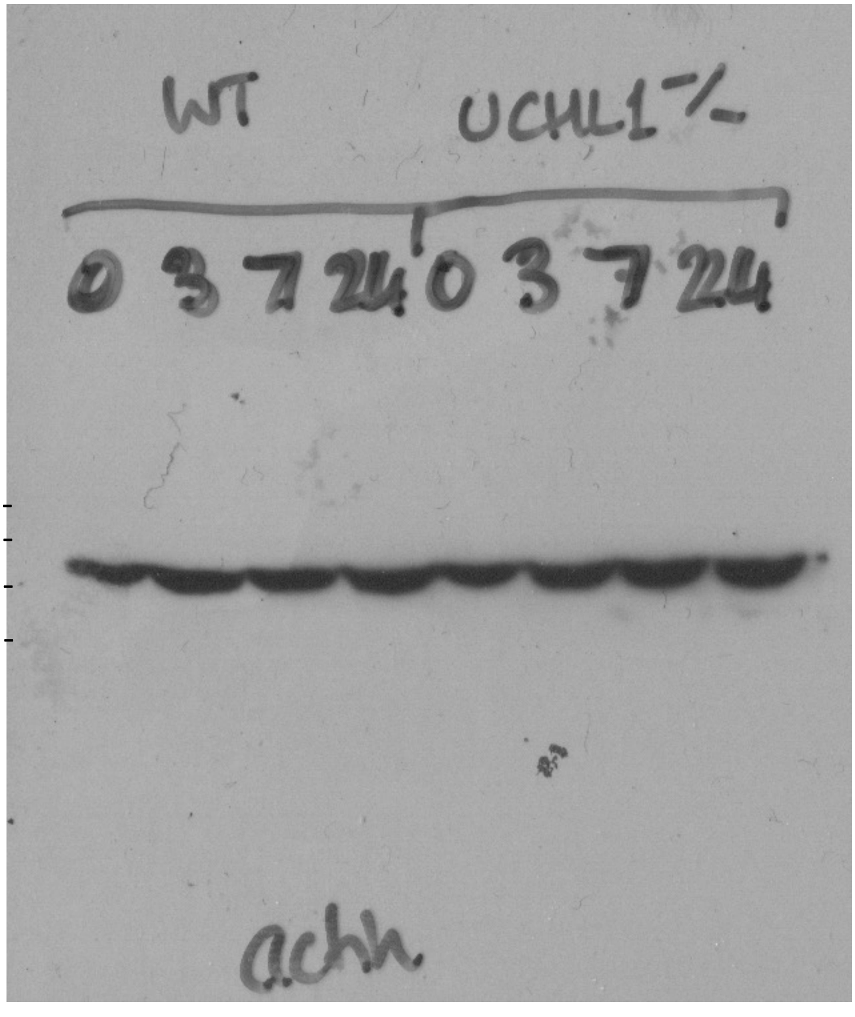

Full Scans
Figure 2D

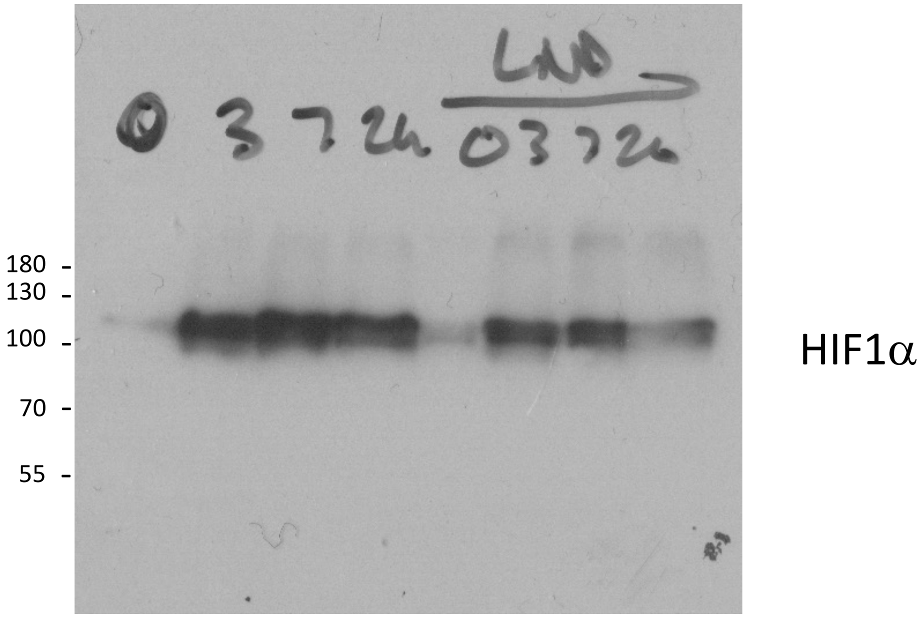

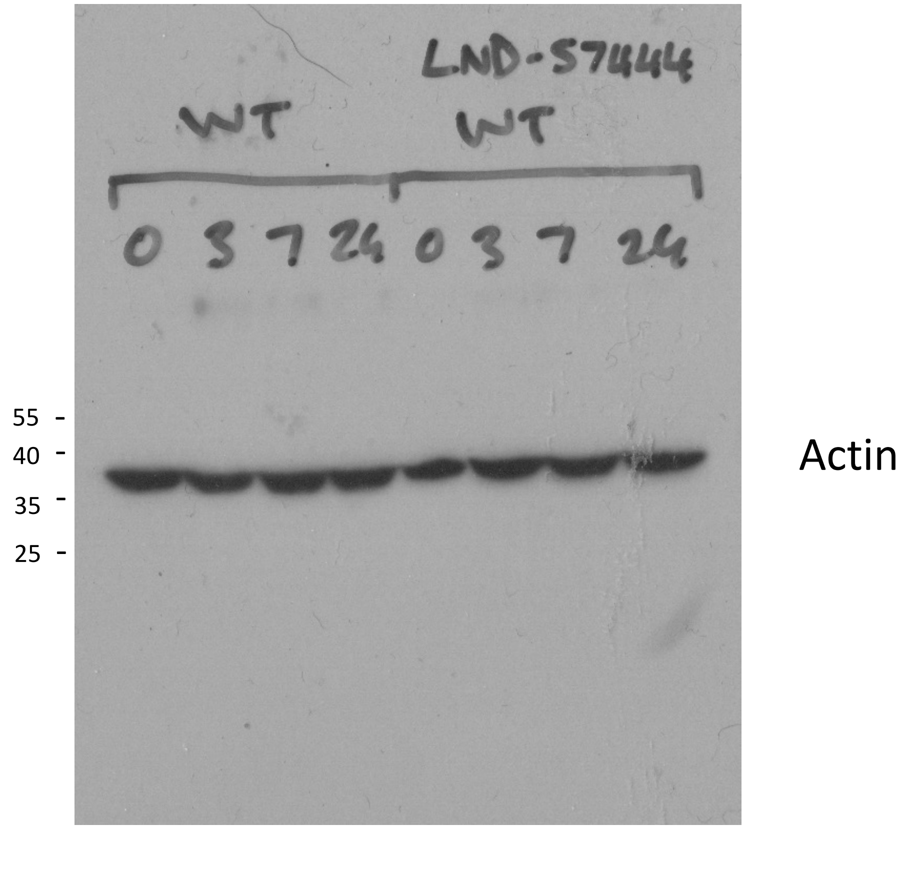

Full Scans
Figure 3A

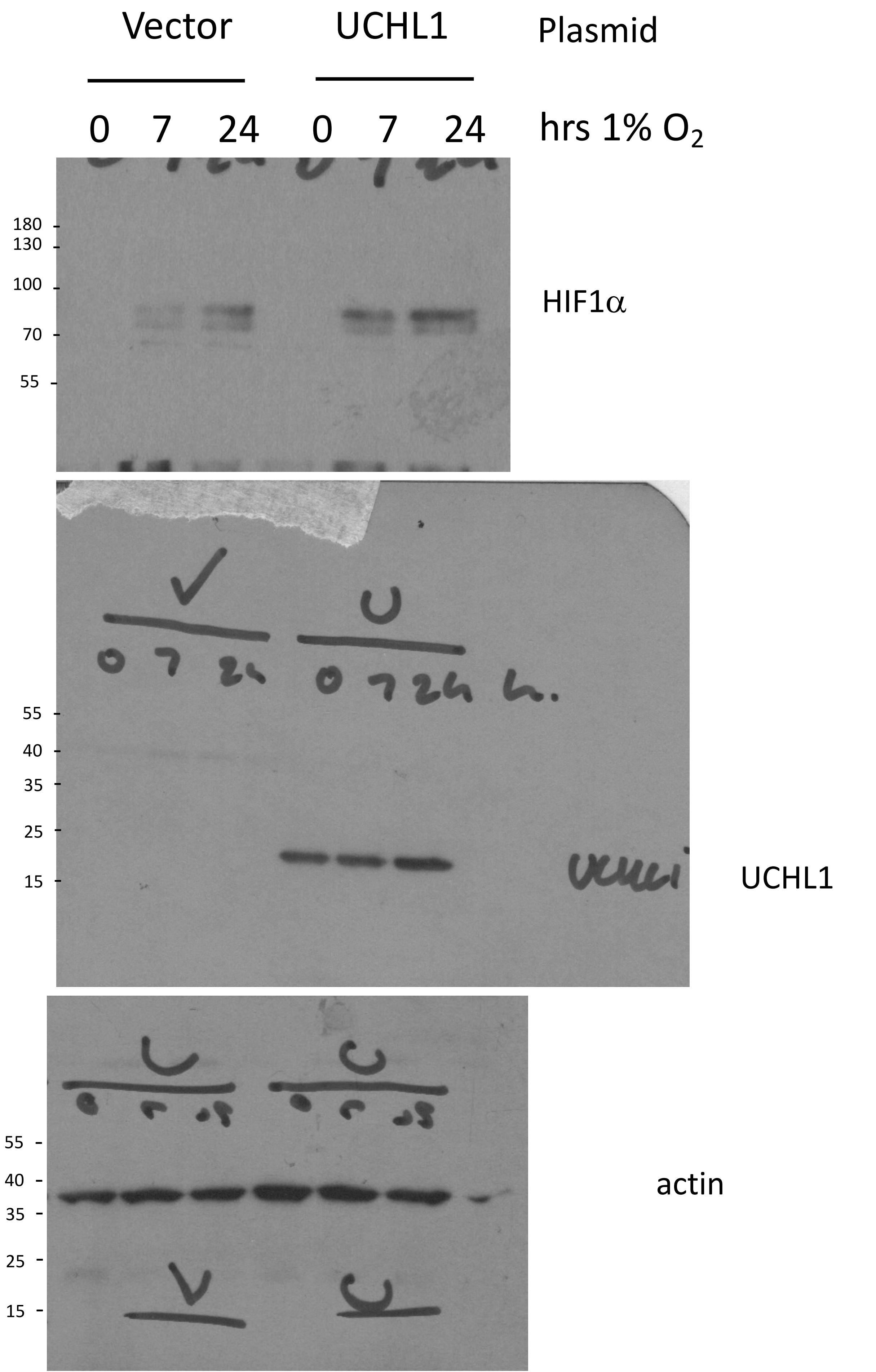

Full Scans
Figure 4A

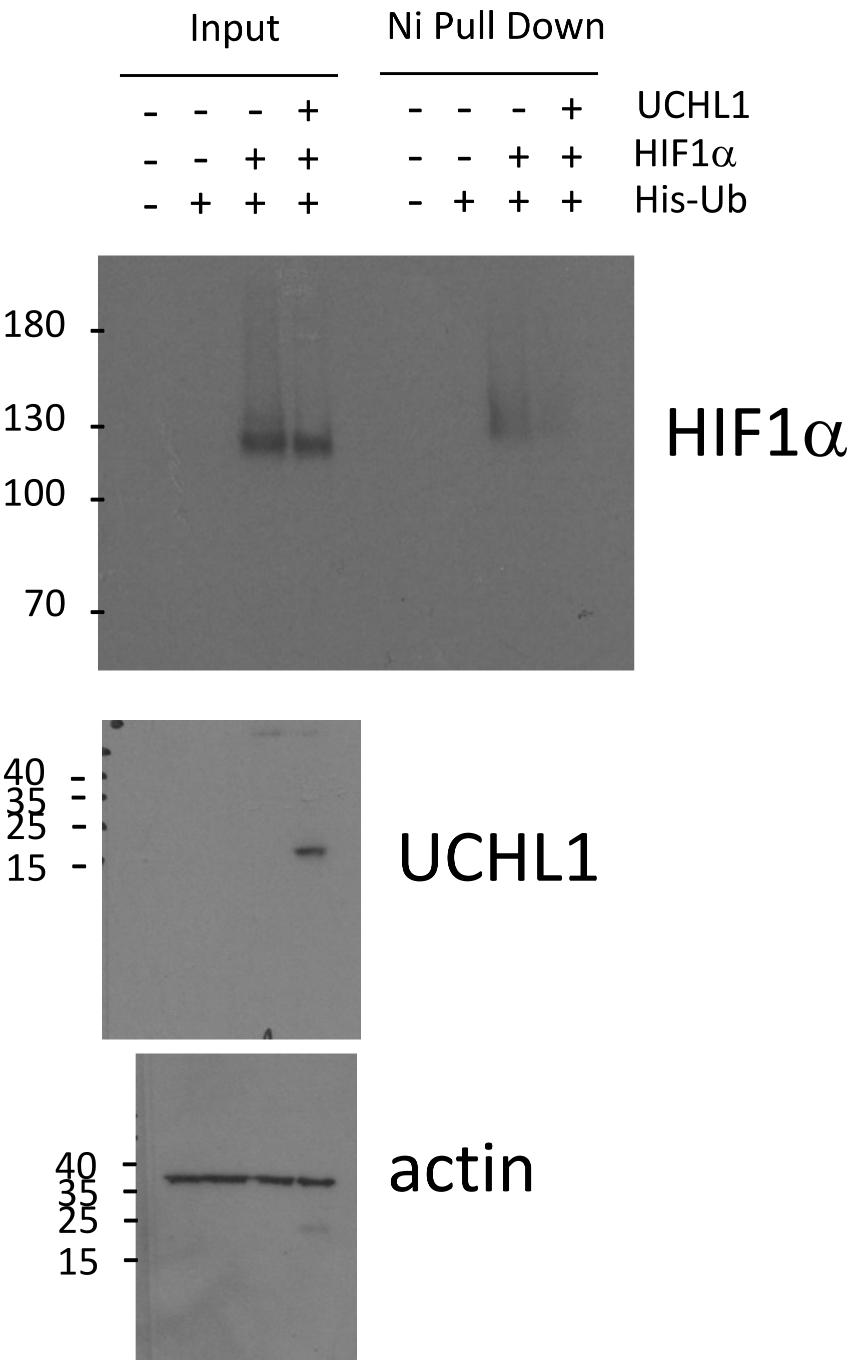

Full Scans
Figure 4B

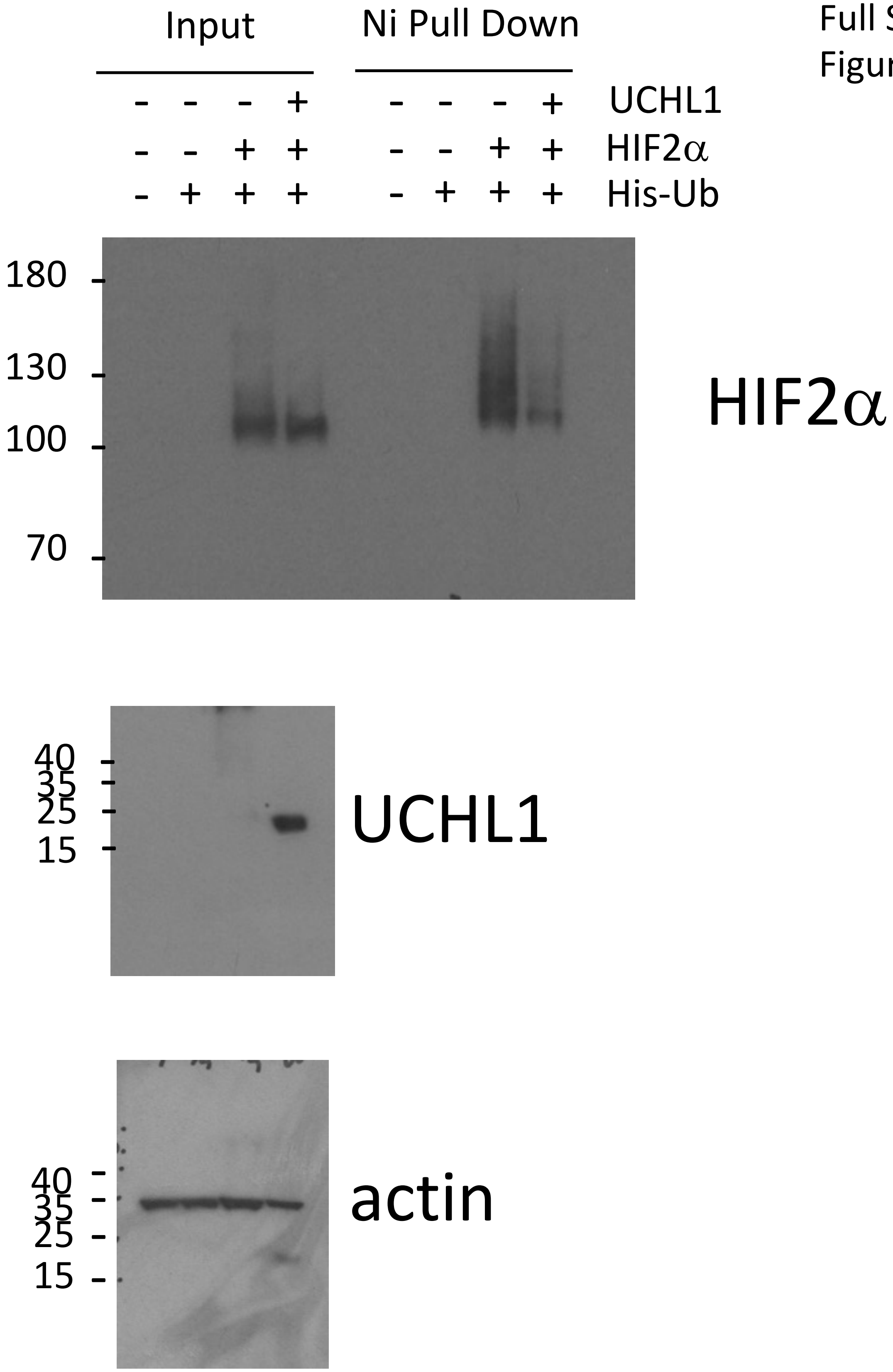

Full Scans
Figure 4C

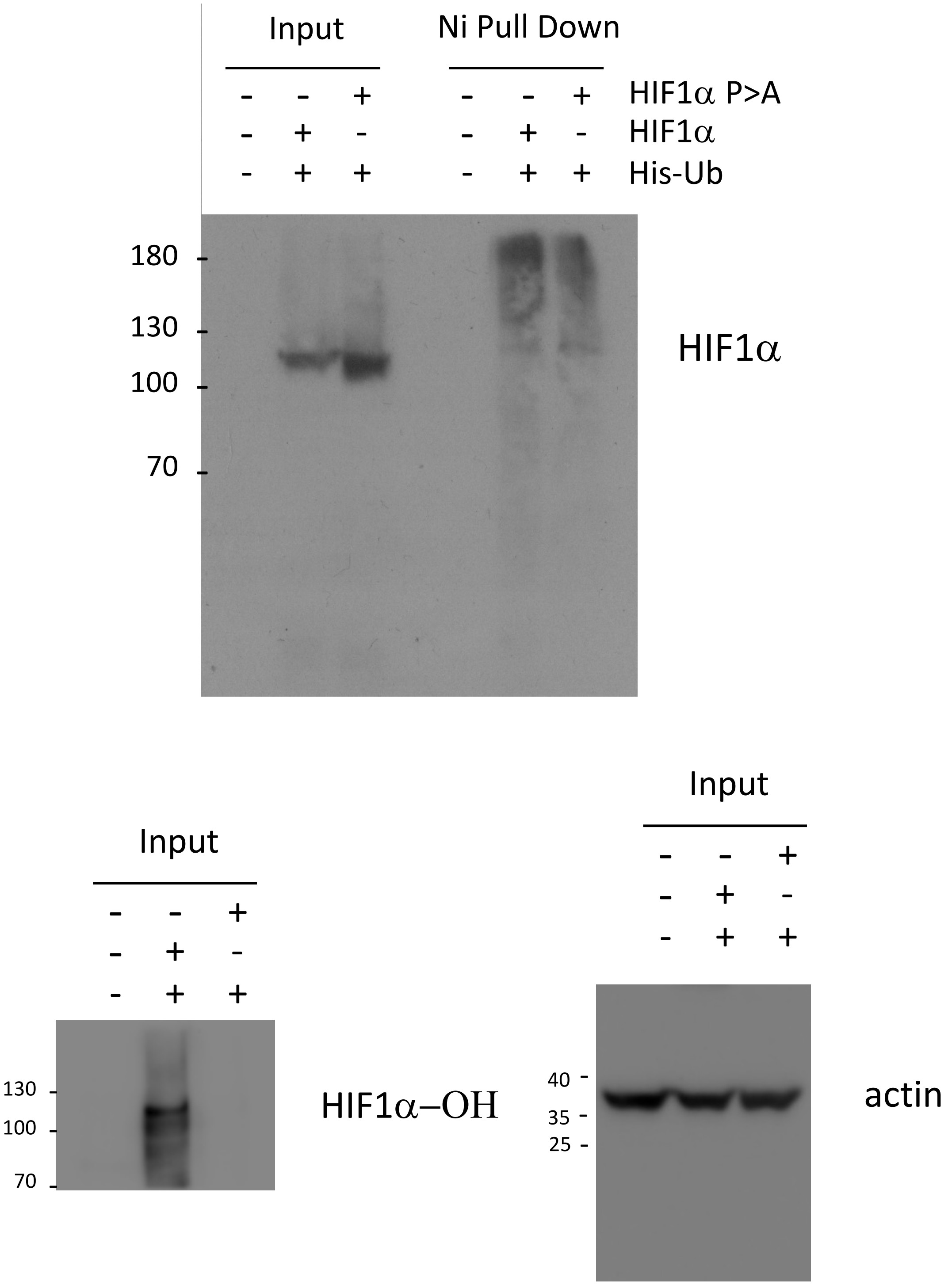

Full Scans
Figure 4D

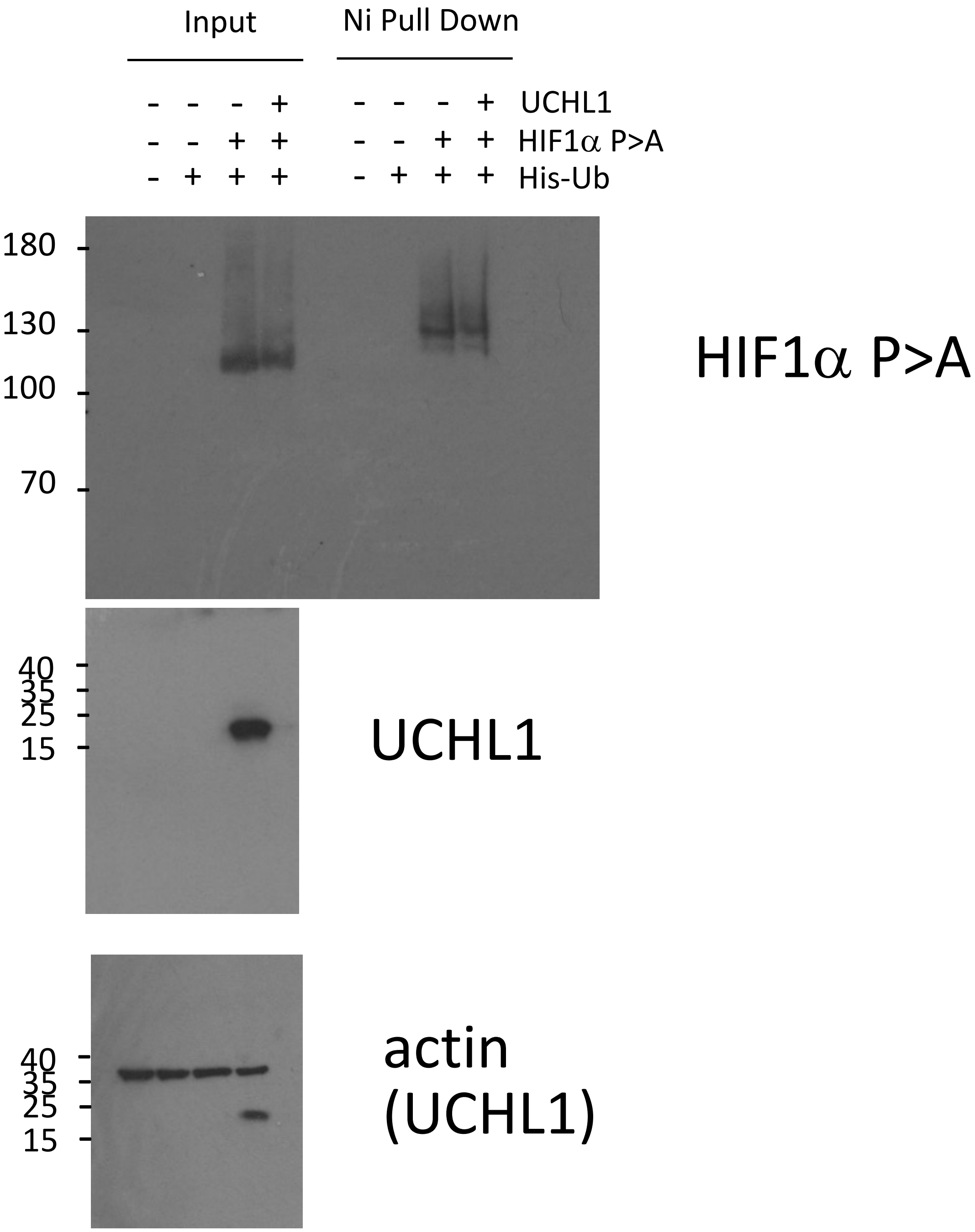

Full Scans
Figure 4E

	Input				Streptavidin Pull Down			
VHL	-	-	+	+	-	-	+	+
UCHL1	-	-	-	+	-	-	-	+
HIF1α	-	+	+	+	-	+	+	+

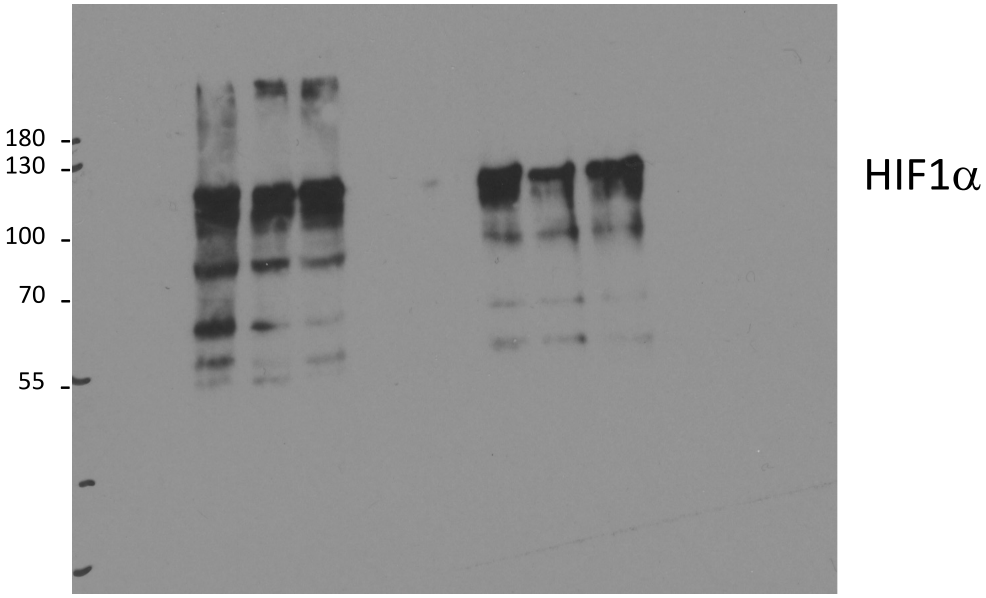

	Input				Streptavidin Pull Down			
VHL	-	-	+	+	-	-	+	+
UCHL1	-	-	-	+	-	-	-	+
HIF1α	-	+	+	+	-	+	+	+

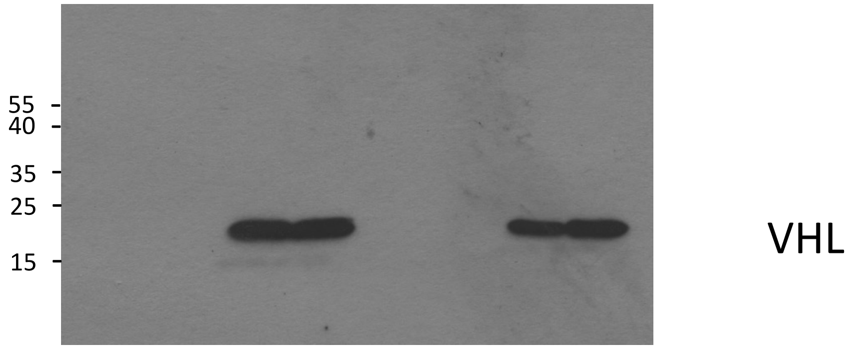

Full Scans
Figure 4E

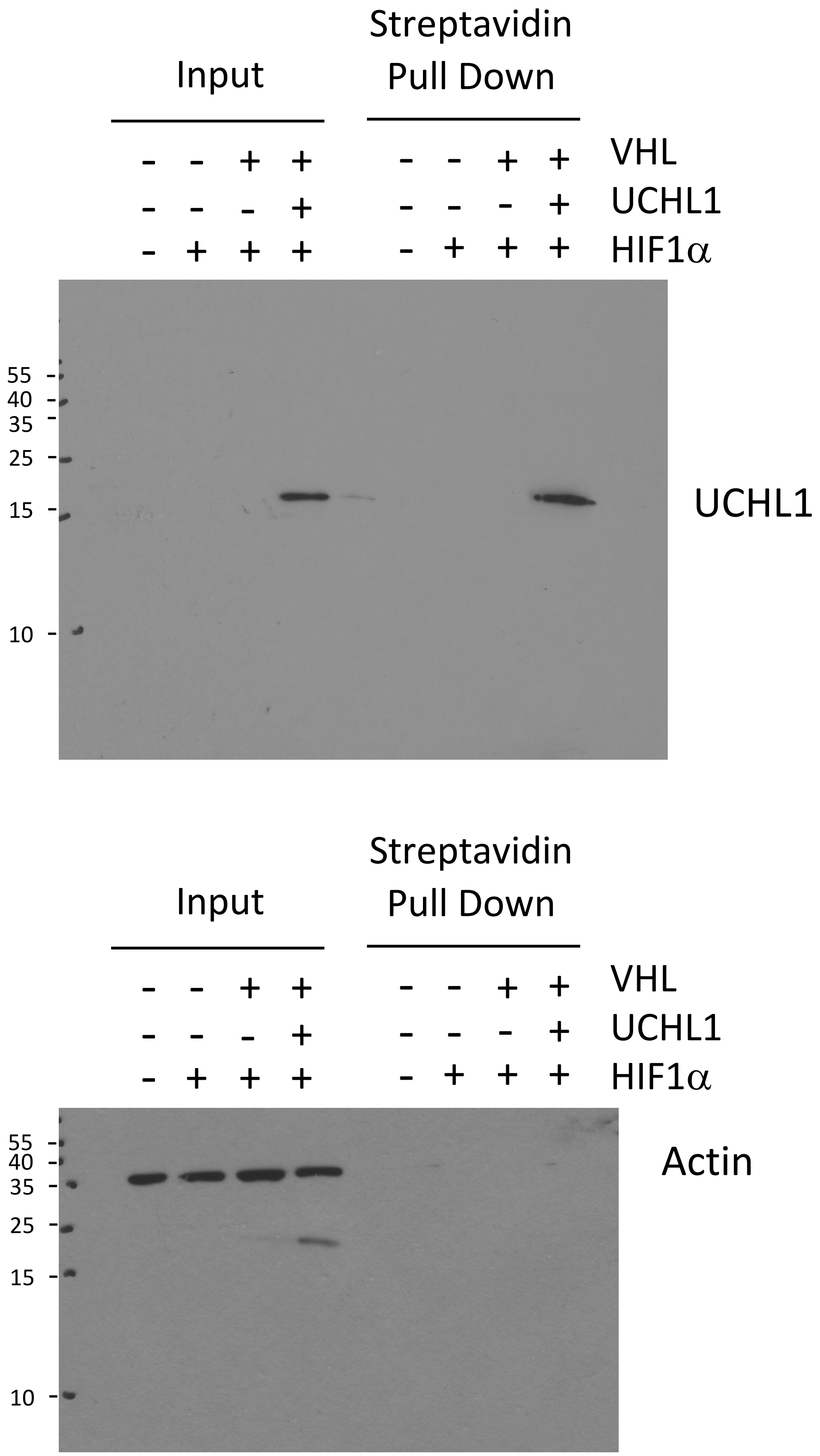

Full Scans
Figure 4F

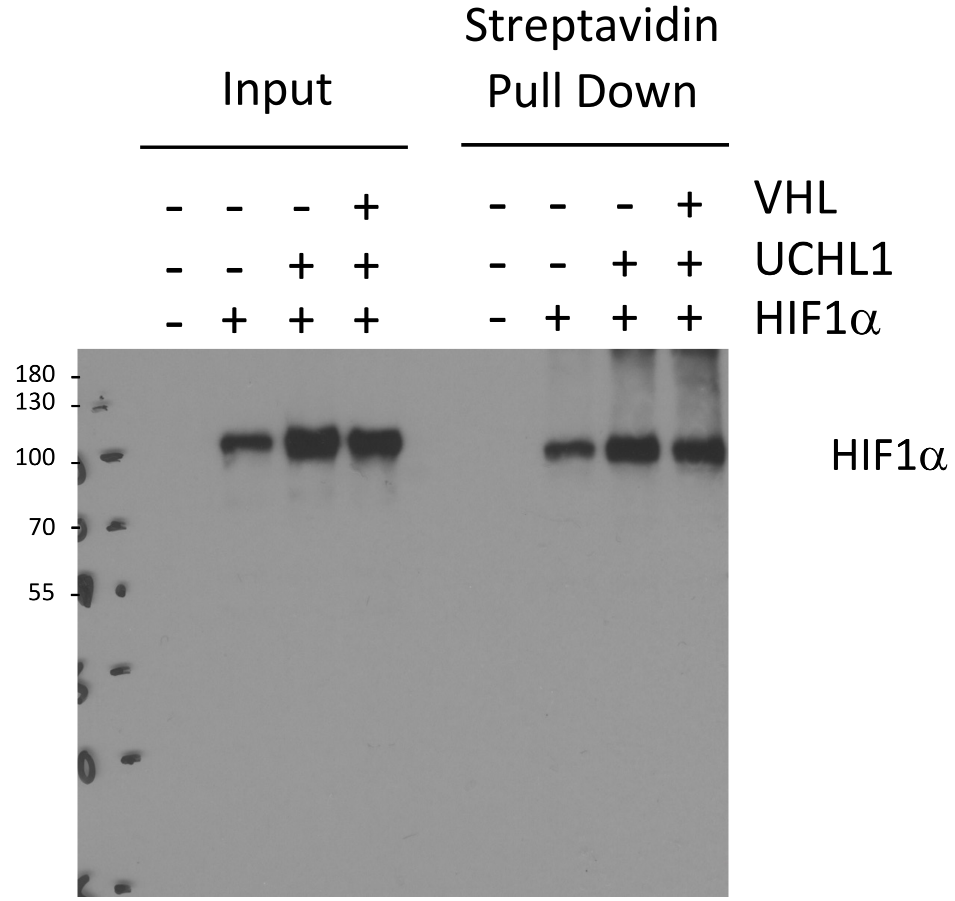

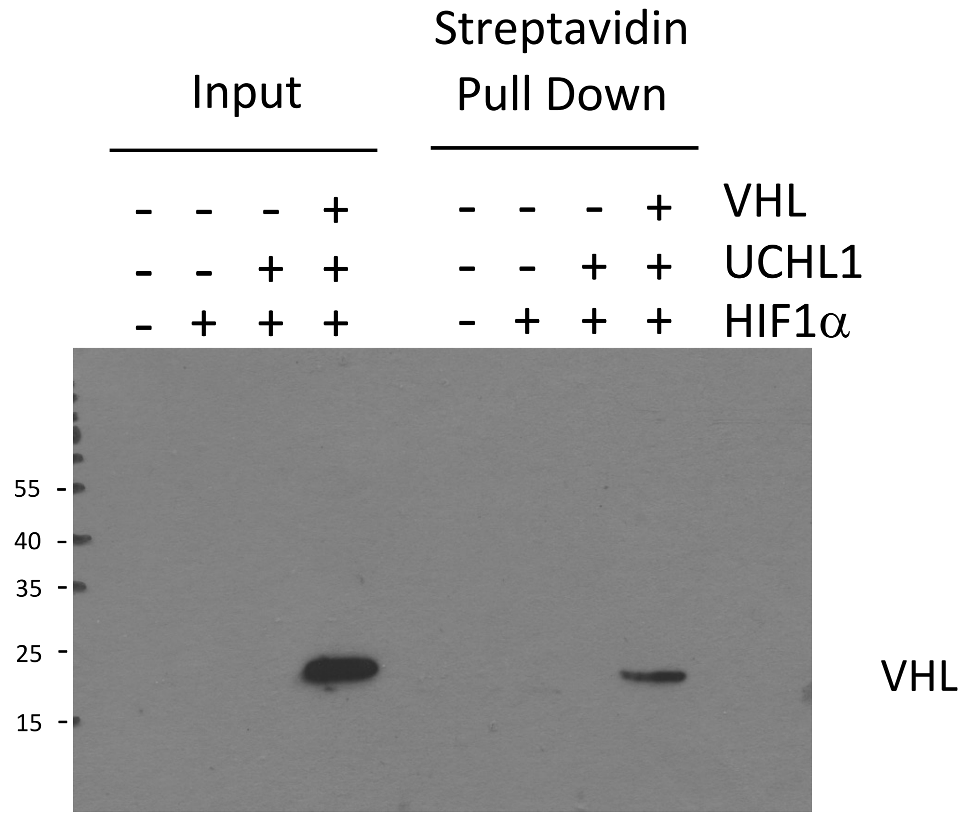

Full Scans
Figure 4F

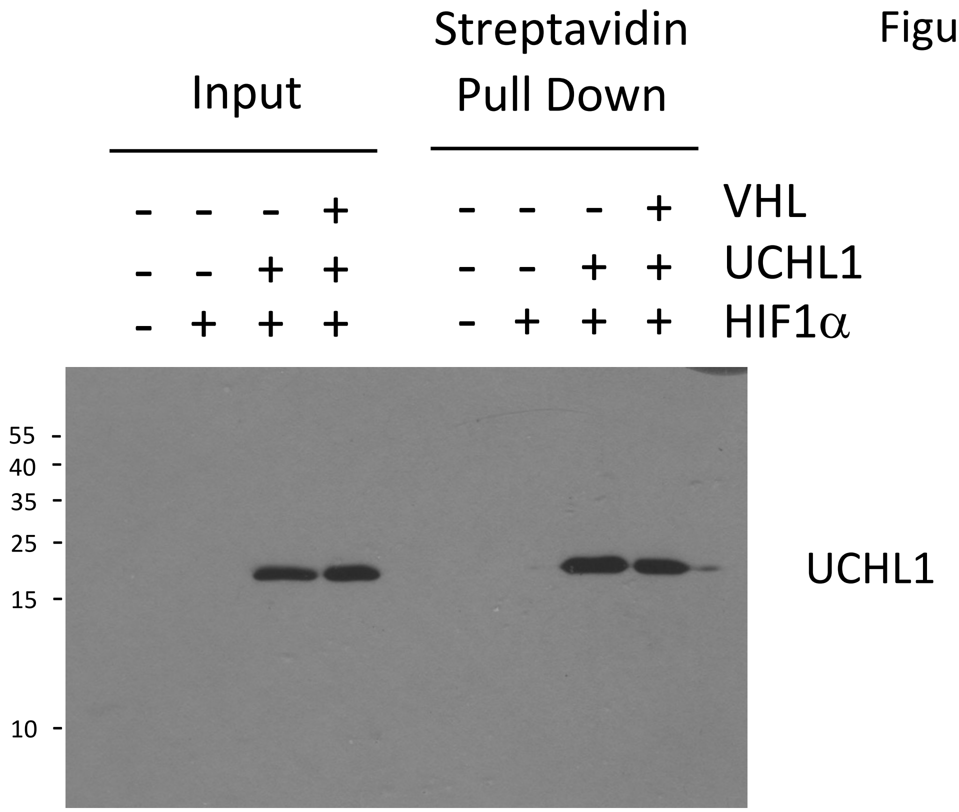

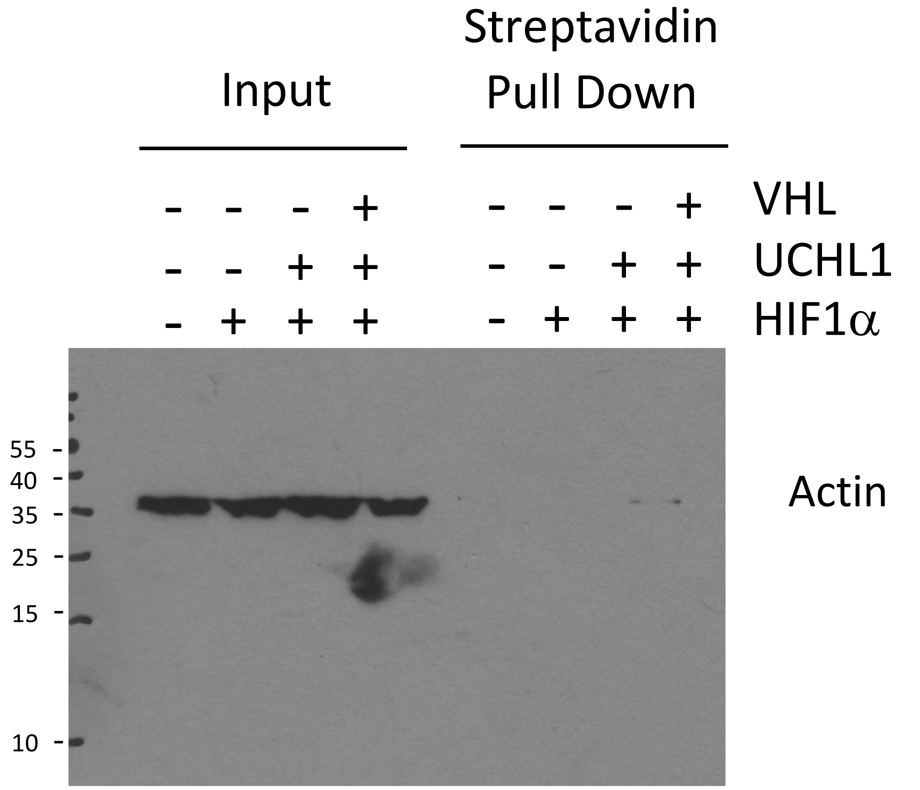

Full Scans
Figure 4G

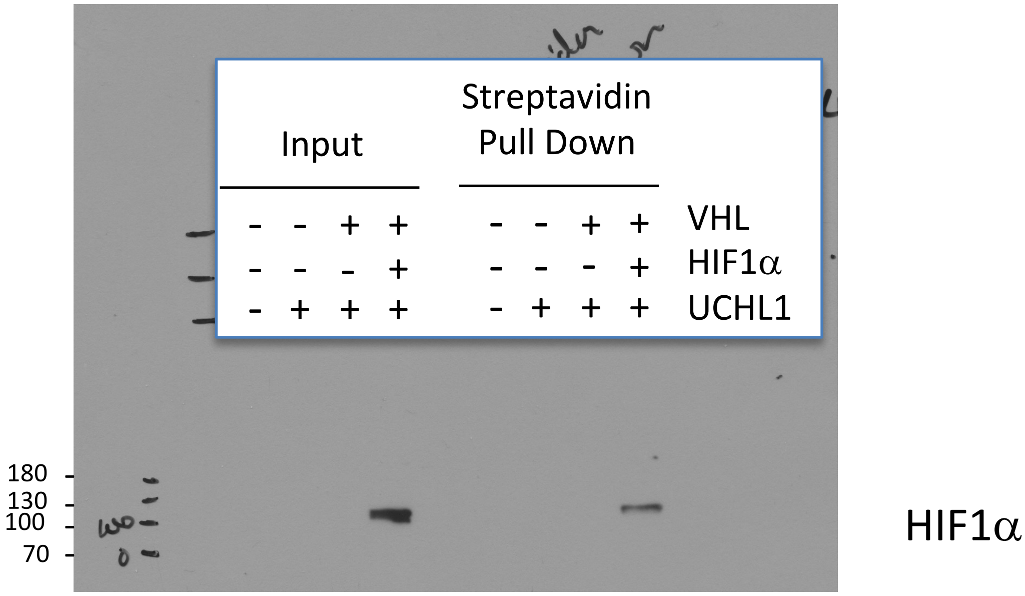

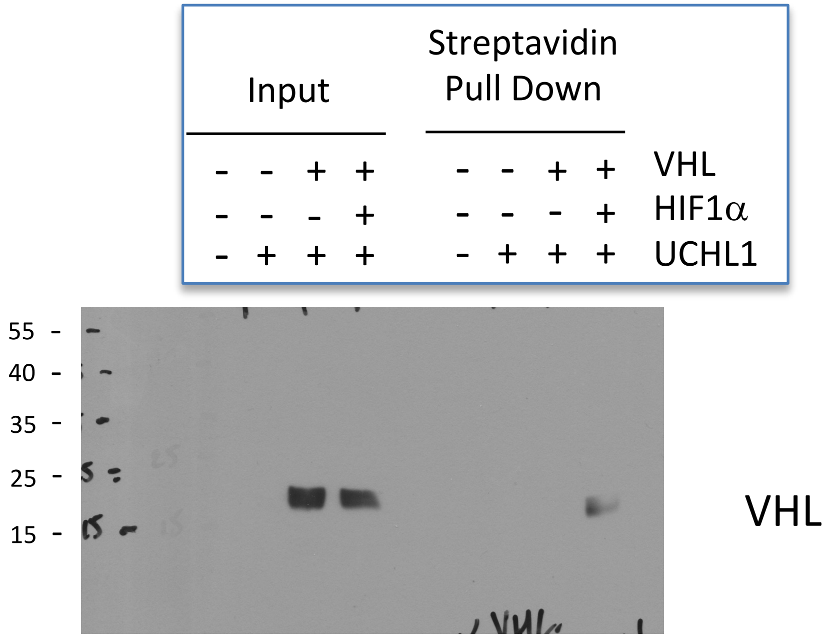

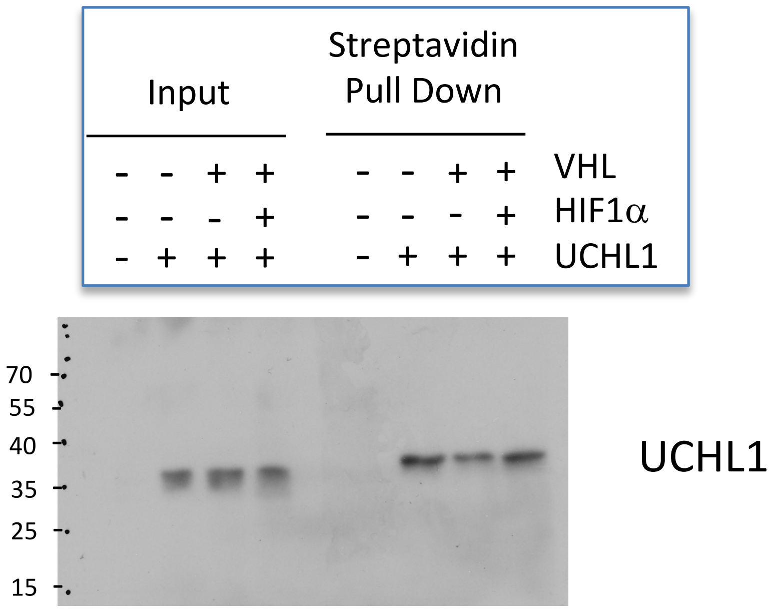
Input
Streptavidin Pull Down
- - + + - - + + VHL
- - - + - - - + HIF1α
- + + + - + + + UCHL1
70
55
40
35
25
15
UCHL1

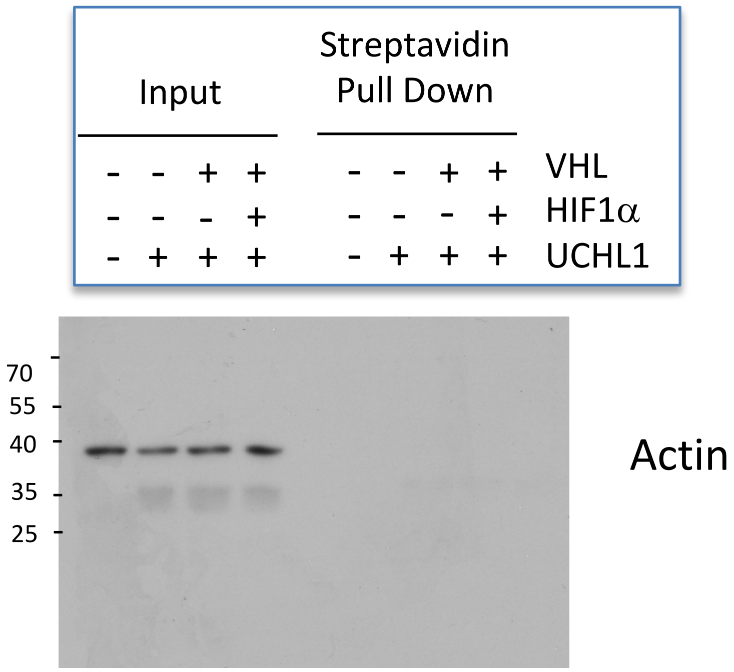
Input
Streptavidin Pull Down
- - + + - - + + VHL
- - - + - - - + HIF1α
- + + + - + + + UCHL1
70
55
40
35
25
Actin

Supplemental Figure 1

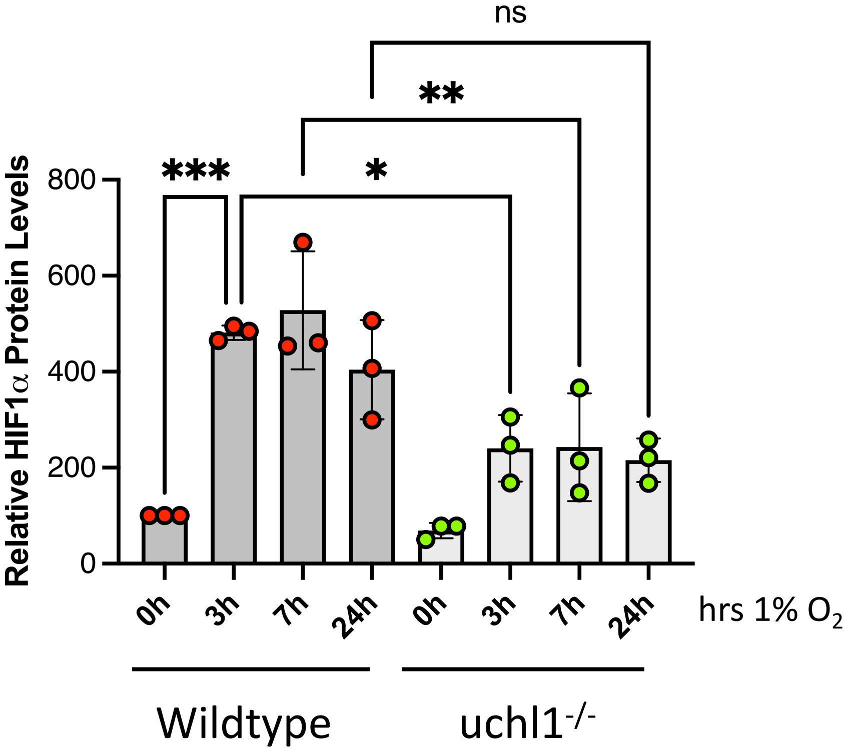

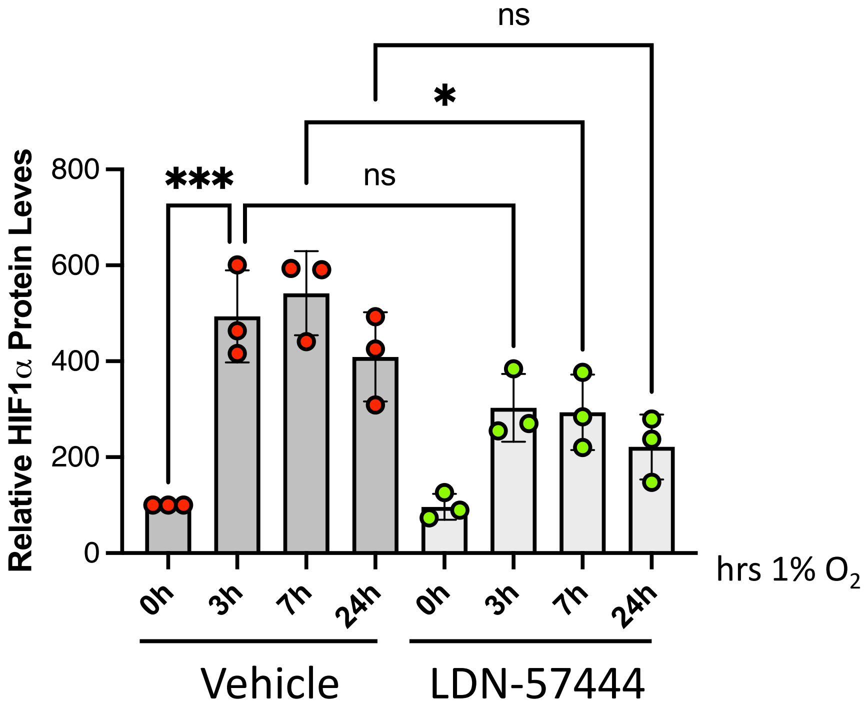

Supplemental Figure 1. HSCs isolated from matched littermate wildtype and uchl1-/- mice exposed to 1% O2 for the indicated times. Whole-cell lysates (WCLs) prepared from these cells were subjected to immunoblot analysis to assess expression levels of the indicated proteins. (D) HSCs prepared from wildtype mice were pre-treated with LDN 57444 (uM) for 30min before being exposed to 1% O2 for the indicated times. Whole-cell lysates (WCLs) prepared from these cells were subjected to immunoblot analysis to assess expression levels of the indicated proteins Relative densitometry of HIF1a immunblot blot bands using ImageJ software. Statistical analysis was performed using GraphPad Prism by a one-way ANOVA with Tukey's multiple comparisons test. If a p-value is less than 0.05, it is flagged with one star (*). If a p-value is less than 0.01, it is flagged with two stars (**). If a p-value is less than 0.001, it is flagged with three stars (***).
